# Antimicrobial Resistance and Infant Mortality in Sri Lanka: A Retrospective Cohort Study

**DOI:** 10.1111/jpc.70269

**Published:** 2026-01-22

**Authors:** Gayana P. S. Gunaratna, Michelle L. Harrison, Benjamin F. R. Dickson, Rajeev Sathanandaraja, T. M. Ruwanthi Perera, Nambage Shirani Chandrasiri, Anasuya Sutharson, Jannah Baker, Phoebe C. M. Williams

**Affiliations:** ^1^ Colombo South Teaching Hospital Colombo Sri Lanka; ^2^ Department of Medical Microbiology Faculty of Medicine, University of Kelaniya Colombo Sri Lanka; ^3^ School of Public Health Faculty of Medicine & Health, The University of Sydney Sydney Australia; ^4^ Sydney Institute for Infectious Diseases (Sydney ID), The University of Sydney Camperdown Australia; ^5^ Department of Paediatrics Faculty of Medical Sciences, University of Sri Jayewardenepura Gangodawila Sri Lanka; ^6^ Department of Infectious Diseases Sydney Children's Hospital Randwick Australia

**Keywords:** epidemiology, neonatal infection, neonatal sepsis, sepsisantimicrobial resistance

## Abstract

**Objective:**

Sepsis is a major cause of mortality among children, with the highest burden evident in neonates and young infants, particularly, in resource‐constrained healthcare settings. Despite this burden, there are insufficient published data to delineate the epidemiology of neonatal sepsis from many of these settings. We aimed to address this research gap by evaluating the epidemiology of sepsis in neonates and young infants in Sri Lanka, a populous country in Southeast Asia, and to evaluate the efficacy of currently‐recommended empiric antibiotic regimens to treat these infections in the context of evolving antimicrobial resistance.

**Design:**

We evaluated the pathogens (including susceptibility profiles) responsible for infections in neonates and young infants over a 7‐year period alongside clinical outcomes (2015–2021).

**Setting:**

A 1100 bed urban tertiary hospital in Colombo, Sri Lanka.

**Patients:**

Neonates and young infants (aged 0 to ≤ 180 days).

**Main Outcome Measures:**

Blood culture‐positive pathogen profiles, antibiotic susceptibility against empiric antibiotic regimens and mortality.

**Results:**

We identified 231 neonates and young infants with clinically significant blood cultures incorporating 251 pathogens over the study period, of whom 22 died. Where gestational data were available, most babies with culture‐positive sepsis were premature (71%, 65/91), born at a median gestational age of 32 weeks (interquartile range [IQR] 27–38 weeks). Gram‐negative bacteria predominated as a cause of culture‐positive infections (66%, 166/251), including in 86% of neonates and young infants who died (19/22). There were high rates of non‐susceptibility to first‐ and second‐line antibiotics currently recommended to treat neonatal sepsis.

**Conclusions:**

There is a high burden of antibiotic‐resistant gram‐negative infections in neonates and young infants in Sri Lanka, highlighting an urgent need to prioritise the development of new antimicrobial regimens to treat neonatal infections.

## Keypoints

- What is already known on this topic
  ○ Neonatal sepsis is highly prevalent in resource‐constrained healthcare settings and is responsible for a rising public health challenge in the context of increasing antimicrobial resistance (AMR) globally.
  ○ Asia is one of the regions with the highest burden of AMR, yet granular data from many resource‐constrained healthcare settings across the region that identify the microbiology and clinical outcomes of culture‐positive infections in neonates and young infants are lacking.
- What this study adds
  ○ Our study describes the significant burden of gram‐negative bacterial infections causative of sepsis in neonates and young infants in an urban tertiary hospital in Sri Lanka. Many of these infections are resistant to antibiotics currently recommended to treat these infections.
  ○ Our data also revealed bacteria traditionally considered as ‘hospital‐acquired’ infections are causing severe, invasive infections in the early neonatal period.
- How this study might affect research, practice or policy
  ○ Our findings align with other recent observational studies that reveal increasingly high rates of AMR in gram‐negative bacterial infections in neonates and young infants.
  ○ Many of the pathogens identified in our study question the traditional definitions (and presumed pathogens responsible for) ‘early’ and ‘late’ onset neonatal sepsis that currently recommended empirical antibiotic regimens propose to cover.
  ○ New antibiotic regimens that provide improved coverage in treating the contemporaneous causes of sepsis in neonates and young infants are urgently needed to reduce the current morbidity and mortality burden due to AMR in this population.

## 1 Introduction

Sepsis is a major cause of neonatal morbidity and mortality [1, 2]. Improvements in global child mortality rates and the attainability of the sustainable development goals depends upon reducing the current burden of neonatal infections. However, neonates are disproportionately affected by the rising threat of antimicrobial resistance (AMR), with one‐third of neonatal sepsis deaths directly attributable to AMR [3, 4, 5, 6, 7].

Despite this mortality burden, there are limited global data on the prevalence of AMR in neonatal sepsis, particularly from resource‐constrained healthcare settings [8, 9]. While high‐income countries have historically identified ‘early onset’ sepsis (EOS) as being caused by pathogens such as Group B Streptococcus (GBS) and *Escherichia coli*, there is mounting evidence to suggest gram‐negative bacteria previously assumed to be nosocomial pathogens (particularly *Klebsiella pneumoniae* and *Acinetobacter baumannii* ) are responsible for the majority of infections in both ‘EOS’ and ‘late onset’ sepsis (LOS) in low‐ and middle‐income countries (LMICs), where the incidence of neonatal sepsis is greatest [8, 10, 11, 12, 13, 14, 15]. This suggests the World Health Organization's (WHO) currently‐recommended empiric first‐ and second‐line antibiotics for treating neonatal sepsis, largely derived from data from high‐income healthcare settings, may be increasingly inefficacious, resulting in high rates of neonatal morbidity and mortality [16, 17, 18].

Gram‐negative bacteria have demonstrated an alarming evolution of multidrug‐resistance in recent years, yet granular data on the incidence and epidemiology of bacteria in high‐burden AMR settings are poorly represented in the literature. Sri Lanka, a lower‐middle income nation in South Asia, has made substantial progress in advancing child and maternal mortality rates over recent decades, largely through the promotion of primary health care [19, 20]. However, recent economic and political instability, combined with rising AMR, threatens to undermine these gains [21].

In 2019, over 8000 deaths in Sri Lanka were associated with multi‐drug‐resistant (MDR) infections, often secondary to hospital outbreaks of Gram‐negative pathogens [22]. While the country has made progress in establishing a national action plan against AMR, multi‐sector collaboration in ensuring its implementation has not yet been developed [22, 23]. As Sri Lanka's health system gains capacity in enabling the survival of premature infants (and those born with congenital anomalies), crowded intensive care units suffering from healthcare worker shortages combined with precarious medication supplies can further promote outbreaks of MDR infections [24].

Neonates and young infants are particularly vulnerable to these risks, given their necessity for prolonged hospitalisation following a premature birth and immature immune systems [25]. Of the 300 000 births occurring each year in Sri Lanka, up to one‐third of these require neonatal intensive care (NICU) or special care admission [26]. Despite national guidelines supporting stringent infection prevention and control programmes in NICU units in Sri Lanka, outbreaks due to MDR‐infections remain a challenge [26].

The WHO have published guidelines for the management of neonatal infections, recommending ampicillin and gentamicin (first‐line) or cefotaxime (second‐line) antibiotics to treat neonatal sepsis [17, 27]. However, these guidelines are historically based on data from high‐income countries that is more readily available (and published), and recommended regimens may not reflect the contemporary epidemiology of neonatal sepsis, particularly in high‐burden AMR settings [15]. Consequently, many hospital settings prescribe broad‐spectrum empiric antibiotics to cover the potential for MDR pathogens, further driving the selection of resistance [28].

The management of neonatal sepsis in high‐burden, resource‐constrained settings is a global health challenge. Despite this, there are limited published clinical and microbiological data informing contemporaneous resistance patterns to guide empirical treatment regimens, particularly from these healthcare settings [29]. By enhancing the availability of robust clinical and microbiological surveillance data on the predominant causative pathogens for neonatal sepsis and their susceptibility profiles, more efficacious empirical antibiotic guidelines may be developed to reduce the current morbidity associated with neonatal sepsis. This study aims to address this research aim by evaluating the epidemiology and clinical outcomes of neonatal sepsis in Sri Lanka, a densely populated country in South Asia [30].

## 2 Methods

### 2.1 Study Design and Setting

We conducted a retrospective cohort study of all culture‐positive bloodstream infections over a seven‐year period (January 2015 to December 2021) at Colombo South Teaching Hospital (CSTH) in Sri Lanka. Due to the retrospective nature of this study, participants were not involved in the design or conduct of the work. We additionally collected available data (vials) on antimicrobial consumption over the same period to broadly evaluate the association of prescribing practices with evolving AMR patterns.

CSTH is a 1110‐bed public tertiary centre in Colombo, the largest city in Sri Lanka. Neonates and infants receive care across an eight‐bed NICU (including one isolation room), a 10‐bed high dependency unit (HDU) (including five isolation cots), a six‐bed special care nursery (SCN), three postnatal wards (90 postnatal beds) and three paediatric wards (90 paediatric beds). The hospital cares for inborn neonates and accepts transfers of out‐born infants requiring higher‐level care with provisions for invasive ventilation, inotropic support and total parenteral nutrition. The unit is staffed by two consultant neonatologists, 10 junior medical officers and 25 nurses who deliver one‐to‐one care during the daytime in addition to a hospital‐wide infection prevention and control (IPC) service.

Physicians at CSTH prescribe empirical antimicrobials for suspected neonatal sepsis according to a combination of the United Kingdom's National Institute for Health and Care Excellence (NICE), WHO guidelines and locally authored guidelines [17, 27, 31]. First‐line treatment recommendations for EOS (defined as sepsis occurring within the first 72 h of life) are benzylpenicillin and gentamicin; for LOS (occurring in infants > 72 h of age) cefotaxime and amikacin are recommended; and the empirical treatment recommendation for neonatal meningitis is cefotaxime.

### 2.2 Study Population

All neonates and young infants (aged 0 to 180 days inclusive) admitted to a neonatal or paediatric ward with clinical sepsis and a positive blood culture collected between January 2015 to December 2021 at CSTH were included. The age range of up to 180 days was selected to ensure extremely premature neonates requiring prolonged hospitalisation would be included in the analysis, given their risk of late‐onset, hospital‐acquired infections that are not often captured in studies focussed on the neonatal period alone. *Corynebacterium* spp., *Micrococcus* spp., *Bacillus* spp. and coagulase‐negative Staphylococci (CoNS) were considered contaminants, as central lines are not inserted within this hospital setting. To confirm these exclusions, where these bacteria were identified, the patient's notes were reviewed to confirm there was no decision to treat the baby with antibiotics due to clinical instability. Additionally, cultures which were unable to be identified beyond microscopy due to microbiology constraints (*n* = 13) were excluded to remove the potential of including further contaminants. Pathogens identified from the same infant within a 4‐week period were considered part of a single infection episode and excluded as duplicates.

### 2.3 Data Sources and Collection

Neonates with positive blood cultures during the study period were identified by systematically reviewing handwritten laboratory records. Available data on collection date and time, specimen type, isolates identified and antimicrobial susceptibility data were extracted. Clinical variables available were also extracted from handwritten clinical notes (Table S1).

During the study period, clinicians collected blood cultures for clinically unstable neonates admitted to the neonatal and paediatric wards. Infants already admitted to those wards also had blood cultures collected before changing antibiotics due to clinical deterioration or to evaluate clearance of known bacteraemia or fungaemia (identified from previously positive cultures).

The CSTH microbiology department analysed all neonatal microbiological tests under the supervision of a consultant microbiologist. Internal quality assurance is undertaken according to CLSI guidelines [32], and external quality assurance is overseen quarterly by the National Microbiology Reference Laboratory Medical Research Institute, Colombo. Blood cultures were processed via a BACTEC automated blood culture system (Becton Dickinson, Sparks USA). Positive cultures subsequently underwent manual identification to the genus level and antimicrobial susceptibility testing was undertaken via phenotypic methods in accordance with CLSI guidelines [33]. For many isolates, the identification of gram‐negative Enterobacterales was limited to lactose‐fermentation status rather than to species level due to laboratory resource constraints. Other gram‐negative isolates requiring identification to the species level were sent to the National Reference Laboratory at the Sri Lankan Medical Research Institute. Alongside these clinical and blood culture data, the number of antimicrobial vials consumed by the neonatal unit for select antimicrobials was extracted from pharmacy records for the study period (Table S2).

### 2.4 Data Analysis

Deidentified data were analysed in R v4.2.1 (R Foundation for Statistical Computing, Austria). The relative frequency of causative pathogens and their associated resistance patterns was calculated, and descriptive statistical analysis evaluated clinical variables associated with infectious episodes.

### 2.5 Ethical Approval

Ethical approval for the study was obtained from the Ethical Review Committee of Colombo South Teaching Hospital, Sri Lanka (approval number 1109).

## 3 Results

### 3.1 Participant Characteristics

The demographic characteristics of 231 infants with 251 significant positive blood cultures over the seven‐year study period are outlined in Table 1. Twenty infants had polymicrobial infections (with two significant pathogens isolated). Most infants were premature (71%, 65/91 of infants with gestational age data available), with a median gestational age of 32 weeks (interquartile range [IQR] 27–38 weeks) and mean birth weight of 1439 g (SD 939 g).

**TABLE 1:** Participant and isolate characteristics.

Characteristic	2015, *N* (%)	2016, *N* (%)	2017, *N* (%)	2018, *N* (%)	2019, *N* (%)	2020, *N* (%)	2021, *N* (%)	Total, *N* (%)
Total infants included	21 (9)	24 (10)	37 (16)	37 (16)	58 (25)	21 (9)	33 (14)	231 (100)
Total positive blood cultures	22 (9)	26 (10)	42 (17)	44 (18)	62 (25)	22 (9)	33 (13)	251 (100)
Pathogen								
Gram‐negative bacteria	13 (59)	13 (50)	25 (60)	29 (66)	45 (73)	14 (64)	27 (82)	166 (66)
Gram‐positive bacteria	9 (41)	9 (35)	10 (24)	9 (20)	15 (24)	7 (32)	5 (15)	64 (25)
Fungi	0 (0)	4 (15)	7 (17)	6 (14)	2 (3)	1 (5)	1 (3)	21 (8)
Gestational age								
*N* (%) with data available	0 (0)	0 (0)	0 (0)	21 (47)	29 (47)	14 (63)	27 (82)	91 (36)
Gestational age (weeks) (median, IQR)	N/A	N/A	N/A	30 (27–38)	33 (30–40)	29 (26–36)	31 (27–35)	32 (27–38)
Birth weight								
*N* (%) with data available	N/A	N/A	N/A	6 (13)	12 (19)	10 (45)	23 (70)	51 (20)
Birth weight (g) (Mean, SD)	N/A	N/A	N/A	1032 (570)	1464 (923)	1517 (1097)	1485 (983)	1433 (940)
Age at onset of sepsis								
*N* (%) with data available	20 (91)	23 (88)	15 (36)	43 (98)	58 (97)	21 (95)	28 (85)	208 (90)
Age (days) at onset of culture‐positive infection (median, IQR)	2 (1–4)	6 (2–11)	4 (2–6)	10 (5–12)	4 (2–7)	5 (4–7)	4 (4–5)	5 (2–10)
Sex								
Female	12 (57)	6 (25)	2 (6)	8 (27)	15 (27)	8 (36)	7 (21)	58 (25)
Male	5 (24)	3 (13)	0 (0)	7 (20)	25 (42)	9 (45)	5 (15)	54 (24)
Missing	4 (19)	15 (63)	35 (95)	22 (53)	18 (31)	4 (18)	21 (64)	119 (51)
Ward location at time of culture‐positive infection								
NICU and SCN	18 (86)	20 (83)	27 (79)	32 (82)	44 (75)	16 (76)	29 (88)	186 (81)
HDU	0 (0)	0 (0)	0 (0)	5 (13)	5 (8)	0 (0)	1 (3)	11 (5)
Postnatal or paediatric ward	3 (14)	4 (17)	7 (21)	2 (5)	10 (17)	5 (24)	3 (9)	34 (15)

The median postnatal age at onset of infection was 5 days (IQR 2–10 days). Most neonates and young infants were admitted to the NICU, HDU or SCN (85%, 197/231) at the time of their infection episode, with only 15% (34/231) admitted to the postnatal or paediatric wards.

### 3.2 Overall Pathogen Frequency

The causative pathogens of 251 infection episodes are summarised in Tables 1 and 2, with gram‐negative bacteria predominating (66%, 166/251, 95% CI 60%–72%), followed by gram‐positive bacteria (25% [64/251], 95% CI 20%–31%) and fungal pathogens (8% [21/251], 95% CI 5%–12%).

**TABLE 2:** Pathogens identified from positive blood culture by early‐ or late‐onset sepsis (*n* = 251).

Species	EOS (0–72 h), *n* (%)	LOS (> 72 h), *n* (%)	Total (excluding those with missing age data)	Total (including those with missing age data)
Gram‐negative pathogens (total)	43 (55)	99 (76)	142 (68)	166 (66)
Enterobacterales (lactose‐fermenting)	13 (17)	50 (38)	63 (30)	66 (26)
Enterobacterales (non‐lactose fermenting)	11 (14)	14 (11)	25 (12)	32 (13)
*Acinetobacter* spp.	15 (19)	31 (24)	46 (22)	49 (20)
*Pseudomonas* spp.	4 (5)	4 (3)	8 (4)	11 (4)
*Haemophilus aphrophilus*	0 (0)	0 (0)	0 (0)	1 (0)
*Rhizobium radiobacter*	0 (0)	0 (0)	0 (0)	7 (3)
Gram‐positive pathogens (total)	35 (45)	16 (12)	51 (25)	64 (25)
*Enterococcus* spp.	3 (4)	4 (3)	7 (3)	9 (4)
Group B Streptococcus	25 (32)	3 (2)	28 (13)	35 (14)
Group A Streptococcus	2 (3)	1 (1)	3 (1)	3 (1)
*Staphylococcus aureus*	3 (4)	8 (6)	11 (5)	14 (6)
*Streptococcus pneumoniae*	2 (3)	0 (0)	2 (1)	3 (1)
Fungal pathogens	0 (0)	15 (12)	15 (7)	21 (8)
*Candida albicans*	0 (0)	3 (2)	3 (1)	3 (1)
*Candida guilliermondii*	0 (0)	1 (1)	1 (1)	2 (1)
*Candida krusei*	0 (0)	1 (1)	1 (1)	1 (0)
*Candida parapsilosis*	0 (0)	5 (4)	5 (2)	9 (4)
Other (or non‐speciated) *Candida* spp.	0 (0)	5 (4)	5 (2)	6 (2)
Total	78 (31)	130 (52)	208 (83)	251 (100)

Among gram‐negative pathogens, lactose‐fermenting coliforms (including *E. coli* and *Klebsiella* spp.) predominated as the main pathogens isolated as causative of sepsis (66/251, 26%); followed by *Acinetobacter* spp. (49/251, 20%) and other non‐lactose‐fermenting coliforms (32/251, 13%).

The most common gram‐positive bacteria causative of neonatal sepsis isolated was GBS (14%, 35/251) followed by *Staphylococcus aureus* (6%, 14/251). *Candida parapsilosis* was the most frequently isolated pathogen causative of fungaemia (9/21, 43%) (Table 2). The study period incorporated a local outbreak of *Rhizobium radiobacter* occurring, cultured in blood from seven clinically unstable preterm infants, as previously described [34].

### 3.3 Frequency by Age

**FIGURE 1:**
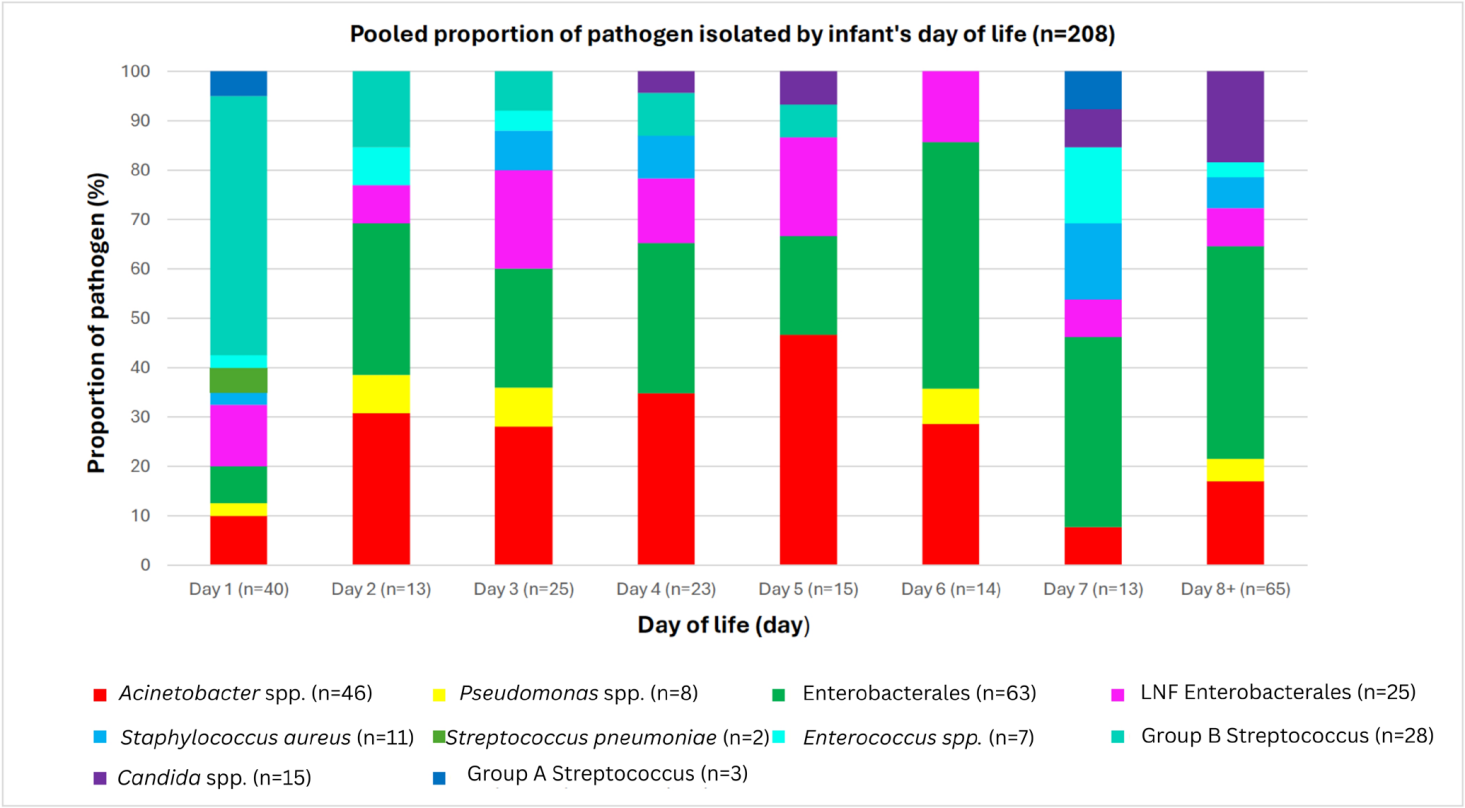
Pooled proportion of isolated pathogens, by day of life. Isolates with missing age of onset have been removed from the graph *n* = 43. LF, lactose fermenting; LNF, lactose non‐fermenting.

**FIGURE 2:**
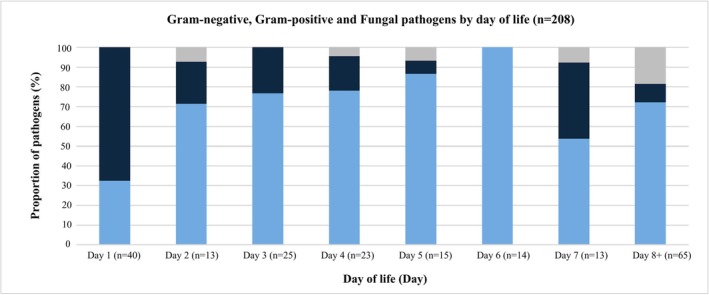
Gram‐negative, gram‐positive and fungal pathogens isolated by neonatal day of life. Isolated pathogens missing age at onset have been excluded from this graph (*n* = 43).

Figures 1 and 2 summarise isolate frequency by postnatal day of life (for 208/251 infants for whom these data were available). Gram‐positive bacteria predominated as a cause of infection on day one (68%, 27/40) with gram‐negative bacteria prevailing subsequently (Figure 2).

Table 2 summarises the relative frequency of isolated pathogens grouped by traditional ‘EOS’ and ‘LOS’ timeframes. Gram‐negative bacteria accounted for 55% (43/78) of EOS episodes. The predominant pathogen isolated in infants with EOS was GBS (32%, 25/78), followed by *Acinetobacter* spp. (19%, 15/78).

Gram‐negative bacteria were responsible for 77% (99/130) of LOS episodes, followed by gram‐positive bacteria (12%, 16/130) and fungal pathogens (11%, 15/130).

### 3.4 Antimicrobial Susceptibility Data

Antimicrobial susceptibility data, including non‐susceptibility to commonly‐prescribed antibiotics by postnatal age, are summarised in Figures S1–S5 and Tables S3 and S4. There was a high proportion of non‐susceptibility evident within gram‐negative bacteria, particularly, in those associated with LOS. Neonates and young infants cared for in the NICU unit had a higher proportion of infections with non‐susceptible bacteria compared with those on the postnatal ward. Non‐susceptibility to third‐generation cephalosporins (ceftriaxone/cefotaxime) was significantly higher for the later years of the study period (2018–2021) when compared to the first years of the study (2015–2017) (Table S4).

### 3.5 Antimicrobial Consumption

Antimicrobial non‐susceptibility and consumption data are summarised in Figure S6. Pearson's correlation (evaluating the association of antibiotic consumption and bacterial non‐susceptibility) revealed no significant association for gentamicin (*r* = −0.34, *p* = 0.578), cefotaxime (*r* = 0.46, *p* = 0.434) or meropenem (*r* = −0.93, *p* = 0.238).

### 3.6 Clinical Outcomes

Due to the limitations in the available clinical (written) records, clinical outcomes were available for neonates and infants admitted in the latter part of the study period only (2018–2021), which enabled the analysis of clinical variables for 64% of enrolled infants (148/231). This excluded seven infants with *Rhizobiom radiobacter* infections in 2017, of whom it has previously been noted that four died [34].

Between 2018 and 2021, 22 infants (of 148, 15%) died due to neonatal sepsis, 86% (19/22) of whom had a Gram‐negative pathogen identified (compared with 14% [3/22] who had a Gram‐positive pathogen identified; Table 3). Of the Gram‐negative bacteria tested for this cohort, 94% (17/18) were non‐susceptible to ampicillin, 89% (16/18) were non‐susceptible to gentamicin (Table 3) and 89% (17/19) were non‐susceptible to meropenem. Most infants who died (55%, 12/22) had MDR infections.

**TABLE 3:** Non‐susceptibility data for inpatient mortality group 2018–2021.

*N* (%) total participants with mortality data available		148/231 (64%)
Inpatient mortality	Alive, *n* (%)	Deceased, *n* (%)
	126 (85)	22 (15)
Pathogen group		
Gram‐negative pathogens	86 (68)	19 (86)
Gram‐positive pathoens	31 (25)	3 (14)
Fungal pathogens	9 (7)	0 (0)

Antimicrobial susceptibility for gram‐negative pathogens										
Antibiotic tested	Ampicillin		Gentamicin		Amikacin		Cefotaxime		Meropenem	
Mortality status	Alive	Deceased	Alive	Deceased	Alive	Deceased	Alive	Deceased	Alive	Deceased
Total tested	64*	18	92	18	94	17	84	19	95	19
Total susceptible	4	1	26	2	57	4	12	1	39	2
% non‐susceptibility	94%	94%	72%	89%	39%	76%	86%	95%	59%	89%

*Note*: 13 infants from the 2018 to 2021 cohort had missing clinical data and have been excluded from analysis.

* *Klebsiella pneumoniae* (*n* = 7) has been removed due to known intrinsic resistance to ampicillin.

## 4 Discussion

This seven year retrospective study of infants with culture‐positive sepsis in Colombo, Sri Lanka, revealed a high prevalence of gram‐negative bacteria causative of sepsis, with concerningly poor coverage provided by the commonly‐recommended empirical antibiotic regimens. This burden of gram‐negative invasive infections in this vulnerable population concurs with other recent studies from LMIC settings and adds important data to the literature from an under‐represented yet high‐burden country that can inform global surveillance studies to enable improved geographic representation in these studies [8, 11, 13, 14, 35].

Our study also notes important novel findings—for example, the proportion of gram‐negative bacteria causative of neonatal sepsis increased over the study period (from 59% in 2015 to 82% in 2021), of concern given the higher likelihood of these bacteria to acquire resistance mechanisms [36, 37]. The clinical impact of this in our cohort is clear, as revealed by the alarming mortality rate in our population, which aligns with similarly concerning mortality rates reported in other recent neonatal sepsis observational studies [38].

Our study also reveals the important role of gram‐negative bacteria in causing both EOS and LOS, including a majority (55%, 43/78) of EOS cases. After day one of life, when gram‐positive pathogens were responsible for two‐thirds of the cases of neonatal sepsis (27/40, 68%), gram‐negative bacteria predominated as a cause of sepsis, with *Acinetobacter* spp.—historically a bacterium associated with nosocomial (late‐onset) infections—the second most prevalent bacteria causative of sepsis in our cohort (46/251, 22%).

These epidemiological shifts in the causative pathogens responsible for neonatal sepsis—and the spread of gram‐negative resistance mechanisms globally—are of significant concern; warranting immediate action to ensure global antibiotic treatment recommendations are updated to reflect the current burden of AMR in neonatal sepsis [39]. The large proportion of gram‐negative bacteria causing sepsis in the early neonatal period in our study, with, particularly, high rates of gram‐negative bacteraemia evident among infants who died as a result of their infection, highlights the need for better empirical treatment regimens to treat neonatal sepsis. Further research is required to enable a clearer understanding of the timing and transmission of bacteria causing neonatal sepsis to determine whether neonates are acquiring (potentially MDR) pathogens vertically during birth, or if they are rapidly becoming colonised with these bacteria within the hospital setting postnatally.

Decision‐making around antimicrobial prescribing is challenging in resource‐constrained settings, as consideration must be given to the clinical presentation of the infant, the likely causative pathogens, access to antimicrobials and the cost of treatment regimens. The challenges of antimicrobial stewardship (AMS) are, particularly, evident in Sri Lanka, where recent economic and political instability has exacerbated shortages of medications and supplies in healthcare institutions [40]. Our data revealed low coverage provided by WHO‐recommended first‐ and second‐line treatment regimens for neonatal sepsis, particularly, in neonates and young infants who died. Resistance to ampicillin and gentamicin exemplifies the challenge of promoting adherence to global guidelines within stewardship programmes, and highlights the need for locally derived and internationally supported empirical regimens with improved efficacy in high burden settings. Meanwhile, there is an urgent need for new antibiotic treatment options to be made available for children and infants, a population in whom research into novel antimicrobial agents (particularly, given their burden of disease) is unacceptably insufficient [25].

High rates of antibiotic prescribing are one of the main drivers of AMR [41], yet in our cohort, we did not find an association between antibiotic consumption and rising resistance within the limitations of the methodology utilised to explore this in our setting. Further investigation is warranted to determine the key drivers of AMR within this setting, including environmental sampling to determine potential sources of gram‐negative bacteria and sequencing to understand transmission dynamics of resistance genes within the NICU, where non‐susceptible infections predominated.

Our findings need to be interpreted within the context of several limitations. First, limited microbiological facilities prevented speciation of some gram‐negative bacteria, impacting species‐specific non‐susceptibility estimates. Second, the paper‐based nature of the local hospital records resulted in some missing data for clinical variables. Furthermore, the inclusion of infants from a single urban centre may bias the result towards more critically unwell infants and overestimate the burden of AMR in Sri Lanka, as regional or community‐based treatment of neonatal infections may respond to more narrow‐spectrum antibiotic agents, such as those recommended by the WHO [17].

Nevertheless, settings such as these are vastly under‐represented in the international literature, and these findings are important to share as calls for updated empirical regimens to treat neonatal sepsis are promoted [14]. Restricted laboratory capacity and clinical staff shortages are problematic in many low‐resourced settings and studies such as ours highlight the need for capacity building—particularly, for AMR surveillance—in this region. Despite the study's limitations, our data have revealed gram‐negative pathogens are dominating the epidemiology of sepsis in hospitalised neonates and young infants in a tertiary urban setting in Sri Lanka, including in the early‐onset neonatal period. Improved stewardship and infection prevention and control programmes will require a deeper understanding of the epidemiology and transmission of neonatal infections. Our study provides strong evidence that further prospective surveillance studies, incorporating both clinical and microbiological data, are clearly needed [29]; and as these data are robustly collated, their contribution to global surveillance networks should prompt the clear need for the development of, and equitable access to, efficacious therapies to treat MDR infections in neonates globally [8, 11, 42].

## Funding

This study was supported by an Australian National Health and Medical Research Council (NHMRC) grant (1197735).

## Ethics Statement

Ethical approval for the study was obtained from the Ethical Review Committee of Colombo South Teaching Hospital, Sri Lanka (approval number 1109).

## Conflicts of Interest

The authors declare no conflicts of interest.

## Supporting information


**Table S1:** Raw data collection tables.

**Table S2:** Antibiotic consumption.

**Table S3:** Antimicrobial susceptibility data table.

**Table S4:** Antimicrobial susceptibility data, aggregated by year.

## Data Availability

The data that supports the findings of this study are available in the Supporting Information of this article.
