# Supplementary material for: Antimicrobial Resistance and Infant Mortality in Sri Lanka: A Retrospective Cohort Study
Source: J Paediatr Child Health. 2026 Jan 22;62(3):446–55. doi: 10.1111/jpc.70269 (PMC12976201; doi:10.1111/jpc.70269)
Supplement: Supplementary file 1 — Table S1: Raw data collection tables. [file JPC-62-446-s004.docx]

| **Supplemental Table 1: Raw data collection tables** |
| --- |

| **Age (days)** | **Year** | **Clinical Syndrome** | **Pathogen** | **Pathogen group** |
| --- | --- | --- | --- | --- |
| 1 | 2020 | meningitis | *S. aureus* | Gram positive bacteria |
| 3 | 2021 | NA | *S. aureus* | Gram positive bacteria |
| 2 | 2021 | NA | LF Coliform | Gram negative bacteria |
| 7 | 2018 | congenital pneumonia | *K. pneumoniae* | Gram negative bacteria |
| NA | 2021 | NA | *Candida* spp*.* | Fungi |
| 14 | 2018 | NA | *Candida parapsilosis* | Fungi |
| 1 | 2021 | NA | NLF Coliform | Gram negative bacteria |
| 28 | 2021 | suspected NEC | *Acinetobacter* spp. | Gram negative bacteria |
| 3 | 2021 | meconium aspiration | LF Coliform | Gram negative bacteria |
| 1 | 2019 | NA | GBS | Gram positive bacteria |
| 8 | 2018 | NA | *S. aureus* | Gram positive bacteria |
| 22 | 2019 | NA | *Pseudomonas* spp. | Gram negative bacteria |
| 3 | 2019 | respiratory distress | *Acinetobacter* spp. | Gram negative bacteria |
| 3 | 2019 | respiratory distress | *Enterobacter cloacae* | Gram negative bacteria |
| 14 | 2018 | sepsis | *E. coli* | Gram negative bacteria |
| 5 | 2020 | NA | *Acinetobacter* spp. | Gram negative bacteria |
| 10 | 2019 | sepsis | *Enterobacter cloacae* | Gram negative bacteria |
| 6 | 2019 | sepsis | *Acinetobacter* spp. | Gram negative bacteria |
| 8 | 2019 | NA | *Pseudomonas* spp. | Gram negative bacteria |
| 2 | 2019 | seizure | LF Coliform | Gram negative bacteria |
| 4 | 2019 | sepsis | NLF Coliform | Gram negative bacteria |
| 6 | 2019 | sepsis | LF Coliform | Gram negative bacteria |
| 2 | 2019 | NA | NLF Coliform | Gram negative bacteria |
| NA | 2019 | NA | Enterococcus sp. | Gram positive bacteria |
| 8 | 2020 | NA | NLF Coliform | Gram negative bacteria |
| 9 | 2020 | NA | LF Coliform | Gram negative bacteria |
| 14 | 2019 | NA | *S. aureus* | Gram positive bacteria |
| 1 | 2019 | NA | *Acinetobacter* spp. | Gram negative bacteria |
| 3 | 2019 | NA | *Acinetobacter* spp. | Gram negative bacteria |
| 11 | 2018 | sepsis | *K. pneumoniae* | Gram negative bacteria |
| 5 | 2019 | sepsis | *Acinetobacter* spp. | Gram negative bacteria |
| 3 | 2019 | meconium aspiration | *Acinetobacter* spp. | Gram negative bacteria |
| 5 | 2020 | NA | *Acinetobacter* spp. | Gram negative bacteria |
| 5 | 2020 | NA | *Acinetobacter* spp. | Gram negative bacteria |
| 5 | 2020 | respiratory distress | *Acinetobacter* spp. | Gram negative bacteria |
| 14 | 2019 | NA | LF Coliform | Gram negative bacteria |
| 2 | 2019 | respiratory distress | *Enterococcus* spp. | Gram positive bacteria |
| NA | 2019 | respiratory distress | *Enterococcus* spp. | Gram positive bacteria |
| 4 | 2019 | sepsis; meningitis | *Acinetobacter* spp. | Gram negative bacteria |
| 1 | 2020 | NA | GBS | Gram positive bacteria |
| 31 | 2019 | periorbital cellulitis | *Enterococcus* spp. | Gram positive bacteria |
| 3 | 2019 | meningitis | NLF Coliform | Gram negative bacteria |
| 1 | 2019 | congenital pneumonia | GBS | Gram positive bacteria |
| 4 | 2020 | sepsis | *Acinetobacter* spp. | Gram negative bacteria |
| 2 | 2019 | NA | *Acinetobacter* spp. | Gram negative bacteria |
| 4 | 2018 | sepsis | S. aureus | Gram positive bacteria |
| 6 | 2019 | meningitis | LF Coliform | Gram negative bacteria |
| 21 | 2018 | sepsis; suspected NEC | *Acinetobacter* spp. | Gram negative bacteria |
| 32 | 2020 | NA | *Candida albicans* | Fungi |
| 13 | 2018 | NA | *Candida albicans* | Fungi |
| 11 | 2019 | NA | *S. aureus* | Gram positive bacteria |
| 3 | 2021 | respiratory distress | GBS | Gram positive bacteria |
| 42 | 2018 | NA | *Candida albicans* | Fungi |
| 9 | 2021 | suspected NEC | *E. coli* | Gram negative bacteria |
| 7 | 2018 | suspected NEC | *Enterococcus faecium* | Gram positive bacteria |
| 6 | 2018 | sepsis | *Enterobacter cloacae* | Gram negative bacteria |
| 1 | 2020 | congenital pneumonia | GBS | Gram positive bacteria |
| 4 | 2021 | sepsis | *E. coli* | Gram negative bacteria |
| 1 | 2020 | birth asphyxia | GBS | Gram positive bacteria |
| 2 | 2021 | sepsis | *E. coli* | Gram negative bacteria |
| 4 | 2020 | sepsis | *S. aureus* | Gram positive bacteria |
| 6 | 2021 | sepsis | NLF Coliform | Gram negative bacteria |
| 10 | 2021 | NA | NLF Coliform | Gram negative bacteria |
| NA | 2021 | NA | NLF Coliform | Gram negative bacteria |
| NA | 2021 | NA NA | NLF Coliform | Gram negative bacteria |
| 4 | 2020 | sepsis; meningitis | *Acinetobacter* spp. | Gram negative bacteria |
| 3 | 2021 | sepsis | NLF Coliform | Gram negative bacteria |
| NA | 2021 | sepsis | NLF Coliform | Gram negative bacteria |
| 11 | 2018 | NA | *Candida parapsilosis* | Fungi |
| 20 | 2018 | sepsis; suspected NEC | *Candida parapsilosis* | Fungi |
| 24 | 2018 | sepsis | *E. faecium* | Gram positive bacteria |
| 11 | 2018 | sepsis | *E. coli* | Gram negative bacteria |
| 11 | 2018 | NA | *K. pneumoniae* | Gram negative bacteria |
| 4 | 2019 | NA | *Acinetobacter* spp. | Gram negative bacteria |
| 4 | 2021 | sepsis | LF Coliform | Gram negative bacteria |
| 3 | 2019 | NA | *Acinetobacter* spp. | Gram negative bacteria |
| 4 | 2018 | sepsis | *Acinetobacter* spp. | Gram negative bacteria |
| 12 | 2018 | sepsis | *Enterobacter cloacae* | Gram negative bacteria |
| 9 | 2021 | sepsis | *S. aureus* | Gram positive bacteria |
| NA | 2018 | congenital pneumonia | GBS | Gram positive bacteria |
| 4 | 2021 | sepsis; meningitis | LF Coliform | Gram negative bacteria |
| 1 | 2019 | birth asphyxia | *Acinetobacter* spp. | Gram negative bacteria |
| 1 | 2021 | NA | NLF Coliform | Gram negative bacteria |
| 31 | 2021 | aspiration pneumonia | LF Coliform | Gram negative bacteria |
| 2 | 2019 | NA | *Pseudomonas* spp. | Gram negative bacteria |
| 14 | 2018 | sepsis | *Enterobacter cloacae* | Gram negative bacteria |
| 6 | 2019 | sepsis | *Acinetobacter* spp. | Gram negative bacteria |
| 12 | 2018 | suspected NEC | NLF Coliform | Gram negative bacteria |
| 1 | 2019 | respiratory distress | GBS | Gram positive bacteria |
| 9 | 2021 | NA | *Acinetobacter* spp. | Gram negative bacteria |
| 2 | 2019 | birth asphyxia | *Acinetobacter* spp. | Gram negative bacteria |
| 1 | 2018 | NA | GBS | Gram positive bacteria |
| 18 | 2018 | NA | *Enterobacter cloacae* | Gram negative bacteria |
| 5 | 2018 | sepsis | LF Coliform | Gram negative bacteria |
| 4 | 2019 | birth asphyxia | *Acinetobacter* spp. | Gram negative bacteria |
| 3 | 2018 | NA | LF Coliform | Gram negative bacteria |
| 7 | 2020 | NA | LF Coliform | Gram negative bacteria |
| 1 | 2019 | NA | GBS | Gram positive bacteria |
| 1 | 2019 | sepsis | *Acinetobacter* spp. | Gram negative bacteria |
| 7 | 2020 | meningitis | *Enterococcus* spp. | Gram positive bacteria |
| 7 | 2021 | sepsis | NLF Coliform | Gram negative bacteria |
| 8 | 2018 | NA | *Acinetobacter baumanii* | Gram negative bacteria |
| 3 | 2021 | NA | *S. aureus* | Gram positive bacteria |
| 1 | 2019 | sepsis | GBS | Gram positive bacteria |
| 1 | 2018 | NA | NLF Coliform | Gram negative bacteria |
| 5 | 2018 | sepsis | *K. pneumoniae* | Gram negative bacteria |
| 7 | 2019 | sepsis | *S. aureus* | Gram positive bacteria |
|  | 2021 | birth asphyxia | *Pseudomonas* spp. | Gram negative bacteria |
| 1 | 2019 | NA | GBS | Gram positive bacteria |
| 4 | 2021 | NA | LF Coliform | Gram negative bacteria |
| 21 | 2018 | NA | *Enterobacter cloacae* | Gram negative bacteria |
| 5 | 2019 | NA | *Acinetobacter baumanii* | Gram negative bacteria |
| 6 | 2021 | NA | LF Coliform | Gram negative bacteria |
| 3 | 2020 | meningitis | LF Coliform | Gram negative bacteria |
| 7 | 2019 | sepsis | *Enterobacter cloacae* | Gram negative bacteria |
| 113 | 2021 | NA | LF Coliform | Gram negative bacteria |
| 1 | 2021 | respiratory distress | *Enterococcus* spp. | Gram positive bacteria |
| 4 | 2019 | NA | NLF Coliform | Gram negative bacteria |
| 33 | 2019 | sepsis | *Acinetobacter* spp. | Gram negative bacteria |
| 7 | 2020 | meningitis | Group A Streptococcus | Gram positive bacteria |
| 10 | 2018 | sepsis | *Candida krusei* | Fungi |
| 14 | 2018 | sepsis | *K. pneumoniae* | Gram negative bacteria |
| 2 | 2021 | convulsion | *Acinetobacter* spp. | Gram negative bacteria |
| 1 | 2018 | NA | *E. Coli* | Gram negative bacteria |
| 9 | 2019 | NA | *K. pneumoniae* | Gram negative bacteria |
| 14 | 2020 | urinary tract infection | *E. Coli* | Gram negative bacteria |
| NA | 2019 | NA | *S. aureus* | Gram positive bacteria |
| 1 | 2018 | meconium aspiration | *Enterobacter aerogenes* | Gram negative bacteria |
| 6 | 2021 | sepsis | LF Coliform | Gram negative bacteria |
| 1 | 2018 | respiratory distress | GBS | Gram positive bacteria |
| 7 | 2019 | NA | *Candida* spp. | Fungi |
| 9 | 2019 | NA | *Enterobacter cloacae* | Gram negative bacteria |
| 3 | 2018 | NA | GBS | Gram positive bacteria |
| 6 | 2018 | sepsis | *Serratia marcescens* | Gram negative bacteria |
| 3 | 2021 | sepsis | NLF Coliform | Gram negative bacteria |
| 4 | 2018 | NA | *Enterobacter cloacae* | Gram negative bacteria |
| 6 | 2018 | sepsis | LF Coliform | Gram negative bacteria |
| 12 | 2018 | NA | *Acinetobacter baumanii* | Gram negative bacteria |
| 7 | 2019 | sepsis | *Enterobacter cloacae* | Gram negative bacteria |
| 5 | 2019 | suspected NEC | NLF Coliform | Gram negative bacteria |
| 3 | 2019 | respiratory distress | *Acinetobacter* spp. | Gram negative bacteria |
| 1 | 2019 | respiratory distress | GBS | Gram positive bacteria |
| 11 | 2018 | NA | *Acinetobacter* spp. | Gram negative bacteria |
| 11 | 2018 | NA | LF Coliform | Gram negative bacteria |
| 11 | 2018 | NA | *Acinetobacter* spp. | Gram negative bacteria |
| 11 | 2018 | sepsis | LF Coliform | Gram negative bacteria |
| 3 | 2019 | meconium aspiration | *Enterobacter cloacae* | Gram negative bacteria |
| 5 | 2019 | NA | NLF Coliform | Gram negative bacteria |
| 16 | 2019 | sepsis | *Serratia marcescens* | Gram negative bacteria |
| 7 | 2020 | sepsis | LF Coliform | Gram negative bacteria |
| 6 | 2020 | suspected NEC | *Pseudomonas* spp*.* | Gram negative bacteria |
| 18 | 2019 | sepsis | *Candida guilliermondii* | Fungi |
| 4 | 2019 | NA | *K. pneumoniae* | Gram negative bacteria |
| NA | 2020 | birth asphyxia | *Pseudomonas* spp*.* | Gram negative bacteria |
| 12 | 2019 | sepsis | *Enterobacter cloacae* | Gram negative bacteria |
| NA | 2019 | respiratory distress | NLF Coliform | Gram negative bacteria |
| 6 | 2019 | NA | *Acinetobacter* spp. | Gram negative bacteria |
| 6 | 2021 | NA | LF Coliform | Gram negative bacteria |
| 3 | 2019 | sepsis | LF Coliform | Gram negative bacteria |
| 3 | 2019 | NA | NLF Coliform | Gram negative bacteria |
| 1 | 2018 | respiratory distress | GBS | Gram positive bacteria |
| NA | 2015 | NA | GBS | Gram positive bacteria |
| 3 | 2015 | NA | NLF Coliforms | Gram negative bacteria |
| 1 | 2015 | NA | GBS | Gram positive bacteria |
| 1 | 2015 | NA | GBS | Gram positive bacteria |
| 1 | 2015 | NA | LF Coliforms | Gram negative bacteria |
| 4 | 2015 | NA | *Acinetobacter* spp. | Gram negative bacteria |
| 5 | 2015 | NA | NLF Coliforms | Gram negative bacteria |
| 30 | 2015 | NA | *Acinetobacter* spp. | Gram negative bacteria |
| 1 | 2015 | NA | NLF Coliforms | Gram negative bacteria |
| 4 | 2015 | NA | NLF Coliforms | Gram negative bacteria |
| 1 | 2015 | NA | GBS | Gram positive bacteria |
| 1 | 2015 | NA | GBS | Gram positive bacteria |
| 4 | 2015 | NA | GBS | Gram positive bacteria |
| 1 | 2015 | NA | Pneumococcus | Gram positive bacteria |
| 1 | 2015 | NA | Group A Streptococcus | Gram positive bacteria |
| 10 | 2015 | NA | *Acinetobacter* spp. | Gram negative bacteria |
| 22 | 2015 | NA | *Acinetobacter* spp. | Gram negative bacteria |
| 3 | 2015 | NA | *Acinetobacter* spp. | Gram negative bacteria |
| 2 | 2015 | NA | *Acinetobacter* spp. | Gram negative bacteria |
| 3 | 2015 | NA | *Acinetobacter* spp. | Gram negative bacteria |
| NA | 2015 | NA | *Acinetobacter* spp. | Gram negative bacteria |
| 1 | 2015 | NA | *Streptococcus pyogenes* | Gram positive bacteria |
| 2 | 2016 | NA | GBS | Gram positive bacteria |
| 9 | 2016 | NA | NLF Coliforms | Gram negative bacteria |
| 7 | 2016 | NA | *Acinetobacter* spp. | Gram negative bacteria |
| 1 | 2016 | NA | GBS | Gram positive bacteria |
| 1 | 2016 | NA | GBS | Gram positive bacteria |
| 1 | 2016 | NA | *Pseudomonas* spp. | Gram negative bacteria |
| NA | 2016 | NA | *Pseudomonas* spp. | Gram negative bacteria |
| NA | 2016 | NA | GBS | Gram positive bacteria |
| 19 | 2016 | NA | LF coliforms | Gram negative bacteria |
| 11 | 2016 | NA | Kleb. Pneumoniae | Gram negative bacteria |
| 15 | 2016 | NA | Enterobacter cloacae | Gram negative bacteria |
| NA | 2016 | NA | GBS | Gram positive bacteria |
| 1 | 2016 | NA | GBS | Gram positive bacteria |
| 10 | 2016 | NA | Candida sp. | Fungi |
| 12 | 2016 | NA | LF coliforms | Gram negative bacteria |
| 15 | 2016 | NA | *Candida* sp. | Fungi |
| 2 | 2016 | NA | GBS | Gram positive bacteria |
| 4 | 2016 | NA | *Enterobacter cloacae* | Gram negative bacteria |
| 1 | 2016 | NA | Pneumococcus | Gram positive bacteria |
| 10 | 2016 | NA | LF coliforms | Gram negative bacteria |
| 5 | 2016 | NA | GBS | Gram positive bacteria |
| 6 | 2016 | NA | *Acinetobacter*. Spp | Gram negative bacteria |
| 12 | 2016 | NA | *Candida* spp. | Fungi |
| 12 | 2016 | NA | *Enterobacter aeroginosa* | Gram negative bacteria |
| 5 | 2016 | NA | *Candida* spp. | Fungi |
| 5 | 2016 | NA | LF coliforms | Gram negative bacteria |
| 4 | 2017 | NA | *Candida parapsilosis* | Fungi |
| 12 | 2017 | NA | *Candida parapsilosis* | Fungi |
| 1 | 2017 | NA | GBS | Gram positive bacteria |
| 3 | 2017 | NA | *Enterococcus* spp. | Gram positive bacteria |
| 3 | 2017 | NA | *Pseudomonas* spp. | Gram negative bacteria |
| 1 | 2017 | NA | NLF Coliform | Gram negative bacteria |
| 1 | 2017 | NA | *Acinetobacter* spp. | Gram negative bacteria |
| 4 | 2017 | NA | *Acinetobacter* spp. | Gram negative bacteria |
| 5 | 2017 | NA | *Acinetobacter* spp. | Gram negative bacteria |
| 8 | 2017 | NA | LF Coliform | Gram negative bacteria |
| 7 | 2017 | NA | MSSA | Gram positive bacteria |
| 2 | 2017 | NA | LF Coliform | Gram negative bacteria |
| 13 | 2017 | NA | *Pseudomonas* spp. | Gram negative bacteria |
| NA | 2017 | NA | NLF Coliform | Gram negative bacteria |
| NA | 2017 | NA | NLF Coliform | Gram negative bacteria |
| NA | 2017 | NA | NLF Coliform | Gram negative bacteria |
| NA | 2017 | NA | LF Coliform | Gram negative bacteria |
| NA | 2017 | NA | *Haemophilus aphrophilus* | Gram negative bacteria |
| NA | 2017 | NA | LF Coliform | Gram negative bacteria |
| NA | 2017 | NA | GBS | Gram positive bacteria |
| NA | 2017 | NA | *Candida parapsilosis* | Fungi |
| NA | 2017 | NA | LF Coliform | Gram negative bacteria |
| 3 | 2017 | NA | *Pseudomonas* spp. | Gram negative bacteria |
| NA | 2017 | NA | MSSA | Gram positive bacteria |
| NA | 2017 | NA | GBS | Gram positive bacteria |
| NA | 2017 | NA | *Acinetobacter* spp. | Gram negative bacteria |
| NA | 2017 | NA | *Rhizobium radiobacter* | Gram negative bacteria |
| NA | 2017 | NA | *Rhizobium radiobacter* | Gram negative bacteria |
| NA | 2017 | NA | *Rhizobium radiobacter* | Gram negative bacteria |
| NA | 2017 | NA | *Candida guilliermondi* | Fungi |
| NA | 2017 | NA | *Candida parapsilosis* | Fungi |
| NA | 2017 | NA | *Rhizobium radiobacter* | Gram negative bacteria |
| NA | 2017 | NA | *Rhizobium radiobacter* | Gram negative bacteria |
| NA | 2017 | NA | *Rhizobium radiobacter* | Gram negative bacteria |
| NA | 2017 | NA | *Rhizobium radiobacter* | Gram negative bacteria |
| NA | 2017 | NA | GBS | Gram positive bacteria |
| NA | 2017 | NA | Pneumococcus | Gram positive bacteria |
| NA | 2017 | NA | *Acinetobacter* spp. | Gram negative bacteria |
| NA | 2017 | NA | MSSA | Gram positive bacteria |
| NA | 2017 | NA | *Candida parapsilosis* | Fungi |
| 4 | 2017 | NA | GBS | Gram positive bacteria |
| NA | 2017 | NA | *Candida parapsilosis* | Fungi |
