# Supplementary material for: Antimicrobial Resistance and Infant Mortality in Sri Lanka: A Retrospective Cohort Study
Source: J Paediatr Child Health. 2026 Jan 22;62(3):446–55. doi: 10.1111/jpc.70269 (PMC12976201; doi:10.1111/jpc.70269)
Supplement: Supplementary file 2 — Table S2: Antibiotic consumption. [file JPC-62-446-s002.docx]

| **Supplementary Table 2. Antibiotic consumption** | | | | | |
| --- | --- | --- | --- | --- | --- |
|  | **Number vials Consumed** | | | | |
| **Antibiotic** | **2016** | **2017** | **2018** | **2019** | **2020** |
| Cefotaxime | 445 | 380 | 300 | 709 | 830 |
| Meropenem | 438 | 274 | 440 | 640 | 99 |
| Gentamicin | 300 | 375 | 300 | 695 | 595 |
