# Supplementary material for: Antimicrobial Resistance and Infant Mortality in Sri Lanka: A Retrospective Cohort Study
Source: J Paediatr Child Health. 2026 Jan 22;62(3):446–55. doi: 10.1111/jpc.70269 (PMC12976201; doi:10.1111/jpc.70269)
Supplement: Supplementary file 3 — Table S3: Antimicrobial susceptibility data table. [file JPC-62-446-s001.docx]

| **Supplementary Table 3: Antimicrobial susceptibility data table** | | | | | | | | | | | | | | | | | | | | | | | | | |
| --- | --- | --- | --- | --- | --- | --- | --- | --- | --- | --- | --- | --- | --- | --- | --- | --- | --- | --- | --- | --- | --- | --- | --- | --- | --- |
| **ID** | **Pathogen** | **Ceft** | **Cefu** | **Gent** | **cipro** | **Neti** | **Aug** | **cotri** | **amik** | **mero** | **eryth** | **clind** | **cefo** | **amp** | **Ceftaz** | **Cef_sul** | **pen** | **col** | **vanc** | **fusi** | **pip** | **lin** | **teico** | **fluco** | **ampho** |
| 4776 | S. aureus | NT | NT | S | R | NT | NT | S | NT | NT | NT | S | S | NT | NT | NT | NT | NT | NT | NT | NT | NT | NT | NT | NT |
| 5479 | S. aureus | NT | NT | R | R | NT | NT | R | NT | NT | R | R | S | NT | NT | NT | NT | NT | NT | NT | NT | NT | NT | NT | NT |
| 9094 | LF Coliform | R | R | R | S | R | R | S | R | R | NT | NT | NT | NT | NT | NT | NT | NT | NT | NT | NT | NT | NT | NT | NT |
| 10779 | K. pneumoniae | NT | R | R | R | R | R | R | R | R | NT | NT | NT | R | R | NT | NT | S | NT | NT | NT | NT | NT | NT | NT |
| 14761 | Candida sp. | NT | NT | NT | NT | NT | NT | NT | NT | NT | NT | NT | NT | NT | NT | NT | NT | NT | NT | NT | NT | NT | NT | NT | NT |
| 17898 | Candida parapsilosis | NT | NT | NT | NT | NT | NT | NT | NT | NT | NT | NT | NT | NT | NT | NT | NT | NT | NT | NT | NT | NT | NT | S | S |
| 20503 | NLF Coliform | S | S | R | S | NT | S | NT | S | S | NT | NT | NT | R | NT | NT | NT | NT | NT | NT | NT | NT | NT | NT | NT |
| 21878 | Acinetobacter sp. | R | R | S | S | R | R | S | S | S | NT | NT | NT | R | NT | NT | NT | NT | NT | NT | NT | NT | NT | NT | NT |
| 21956 | LF Coliform | S | S | S | S | R | S | NT | S | S | NT | NT | NT | R | NT | NT | NT | NT | NT | NT | NT | NT | NT | NT | NT |
| 22157 | GBS | NT | NT | NT | NT | NT | NT | NT | NT | NT | S | S | NT | S | NT | NT | S | NT | NT | NT | NT | NT | NT | NT | NT |
| 22672 | S. aureus | NT | NT | R | R | NT | NT | NT | NT | NT | R | R | R | NT | NT | NT | NT | NT | S | S | NT | NT | NT | NT | NT |
| 22768 | Pseudomonas sp. | NT | NT | R | R | R | R | R | R | S | NT | NT | NT | NT | S | NT | NT | NT | NT | NT | NT | NT | NT | NT | NT |
| 23419 | Acinetobacter sp. | R | NT | R | R | R | R | R | NT | R | NT | NT | NT | R | R | NT | NT | NT | NT | NT | NT | NT | NT | NT | NT |
| 23780 | Enterobacter cloacae | R | NT | R | R | NT | R | R | S | R | NT | NT | NT | NT | R | NT | NT | NT | NT | NT | NT | NT | NT | NT | NT |
| 25744 | E. Coli | R | R | R | R | NT | R | NT | S | S | NT | NT | NT | R | NT | NT | NT | NT | NT | NT | S | NT | NT | NT | NT |
| 25830 | Acinetobacter sp. | NT | NT | R | R | NT | R | R | R | R | NT | NT | NT | NT | R | R | NT | NT | NT | NT | NT | NT | NT | NT | NT |
| 27765 | Enterobacter cloacae | S | NT | S | S | S | R | S | S | S | NT | NT | NT | R | S | NT | NT | NT | NT | NT | NT | NT | NT | NT | NT |
| 31585 | Acinetobacter sp. | R | R | R | R | R | R | R | R | R | NT | NT | NT | NT | R | NT | NT | NT | NT | NT | NT | NT | NT | NT | NT |
| 31585 | Pseudomonas sp. | NT | NT | R | R | R | NT | R | R | R | NT | NT | NT | NT | R | NT | NT | NT | NT | NT | NT | NT | NT | NT | NT |
| 32268 | LF Coliform | R | R | R | R | R | R | R | NT | S | NT | NT | NT | R | NT | NT | NT | NT | NT | NT | NT | NT | NT | NT | NT |
| 32330 | NLF Coliform | S | S | S | S | S | S | S | NT | S | NT | NT | NT | S | S | NT | NT | NT | NT | NT | NT | NT | NT | NT | NT |
| 32728 | LF Coliform | R | R | R | R | R | R | R | S | R | NT | NT | NT | R | R | NT | NT | NT | NT | NT | NT | NT | NT | NT | NT |
| 33793 | NLF Coliform | R | R | R | R | R | R | R | R | R | NT | NT | NT | R | R | NT | NT | NT | NT | NT | NT | NT | NT | NT | NT |
| 34454 | Enterococcus sp. | NT | NT | NT | NT | NT | NT | NT | NT | NT | R | R | NT | S | NT | NT | S | NT | NT | NT | NT | NT | NT | NT | NT |
| 34567 | NLF Coliform | R | R | R | R | NT | R | NT | S | R | NT | NT | NT | R | R | NT | NT | NT | NT | NT | NT | NT | NT | NT | NT |
| **ID** | **Pathogen** | **Ceft** | **Cefu** | **Gent** | **cipro** | **Neti** | **Aug** | **cotri** | **amik** | **mero** | **eryth** | **clind** | **cefo** | **amp** | **Ceftaz** | **Cef_sul** | **pen** | **col** | **vanc** | **fusi** | **pip** | **lin** | **teico** | **fluco** | **ampho** |
| 34567 | LF Coliform | R | R | R | R | NT | R | R | S | R | NT | NT | NT | NT | NT | NT | NT | NT | NT | NT | NT | NT | NT | NT | NT |
| 34655 | S. aureus | NT | NT | S | S | NT | NT | S | NT | NT | R | R | S | NT | NT | NT | R | NT | NT | NT | NT | NT | NT | NT | NT |
| 35088 | Acinetobacter sp. | R | R | R | R | R | R | NT | R | R | NT | NT | NT | R | R | NT | NT | NT | NT | NT | NT | NT | NT | NT | NT |
| 35226 | Acinetobacter sp. | R | R | R | R | S | R | S | S | S | NT | NT | NT | R | R | NT | NT | NT | NT | NT | NT | NT | NT | NT | NT |
| 36139 | K. pneumoniae | R | R | R | R | R | R | S | R | R | NT | NT | NT | R | R | NT | NT | NT | NT | NT | NT | NT | NT | NT | NT |
| 37476 | Acinetobacter sp. | R | NT | R | R | NT | R | R | R | R | NT | NT | NT | R | R | NT | NT | NT | NT | NT | NT | NT | NT | NT | NT |
| 38007 | Acinetobacter sp. | R | R | R | R | NT | NT | R | R | R | NT | NT | NT | R | R | NT | NT | NT | NT | NT | NT | NT | NT | NT | NT |
| 39198 | Acinetobacter sp. | R | NT | R | R | NT | R | R | R | R | NT | NT | NT | R | R | NT | NT | NT | NT | NT | NT | NT | NT | NT | NT |
| 39199 | Acinetobacter sp. | R | NT | R | R | NT | R | R | R | R | NT | NT | NT | R | R | NT | NT | NT | NT | NT | NT | NT | NT | NT | NT |
| 39200 | Acinetobacter sp. | R | NT | S | S | NT | S | S | S | S | NT | NT | NT | R | R | NT | NT | NT | NT | NT | NT | NT | NT | NT | NT |
| 39256 | LF Coliform | S | S | S | NT | S | S | S | S | S | NT | NT | NT | S | S | NT | NT | NT | NT | NT | NT | NT | NT | NT | NT |
| 39664 | Enterococcus sp. | NT | NT | NT | NT | NT | NT | NT | NT | NT | R | R | NT | S | NT | NT | S | NT | NT | NT | NT | NT | NT | NT | NT |
| 40008 | Enterococcus sp. | NT | NT | NT | NT | NT | NT | NT | NT | NT | R | R | NT | S | NT | NT | S | NT | NT | NT | NT | NT | NT | NT | NT |
| 41144 | Acinetobacter sp. | R | R | R | NT | R | R | R | R | R | NT | NT | NT | NT | R | NT | NT | NT | NT | NT | NT | NT | NT | NT | NT |
| 41607 | GBS | NT | NT | NT | NT | NT | NT | NT | NT | NT | NT | NT | NT | S | NT | NT | S | NT | NT | NT | NT | NT | NT | NT | NT |
| 43117 | Enterococcus sp. | NT | NT | NT | NT | NT | NT | NT | NT | NT | R | S | NT | S | NT | NT | S | NT | NT | NT | NT | NT | NT | NT | NT |
| 44317 | NLF Coliform | S | NT | S | NT | S | R | NT | S | R | NT | NT | NT | R | S | NT | NT | NT | NT | NT | NT | NT | NT | NT | NT |
| 44615 | GBS | NT | NT | NT | NT | NT | NT | NT | NT | NT | R | R | NT | S | NT | NT | S | NT | NT | NT | NT | NT | NT | NT | NT |
| 44718 | Acinetobacter sp. | R | NT | R | R | NT | R | R | R | R | NT | NT | NT | R | R | NT | NT | NT | NT | NT | NT | NT | NT | NT | NT |
| 44749 | Acinetobacter sp. | R | R | S | S | S | R | NT | S | S | NT | NT | NT | R | NT | NT | NT | NT | NT | NT | NT | NT | NT | NT | NT |
| 45019 | S. aureus | NT | NT | S | S | NT | NT | S | NT | NT | S | S | S | NT | NT | NT | NT | NT | NT | NT | NT | NT | NT | NT | NT |
| 45098 | LF Coliform | S | R | S | S | S | R | S | S | S | NT | NT | NT | R | S | NT | NT | NT | NT | NT | NT | NT | NT | NT | NT |
| 45120 | Acinetobacter sp. | R | R | R | R | R | R | S | R | R | NT | NT | NT | R | NT | S | NT | NT | NT | NT | NT | NT | NT | NT | NT |
| 46128 | Candida albicans | NT | NT | NT | NT | NT | NT | NT | NT | NT | NT | NT | NT | NT | NT | NT | NT | NT | NT | NT | NT | NT | NT | S | NT |
| 46569 | Candida albicans | NT | NT | NT | NT | NT | NT | NT | NT | NT | NT | NT | NT | NT | NT | NT | NT | NT | NT | NT | NT | NT | NT | S | S |
| 47316 | S. aureus | NT | NT | S | S | NT | NT | S | NT | NT | S | S | S | NT | NT | NT | R | NT | NT | NT | NT | NT | NT | NT | NT |
| 48673 | GBS | NT | NT | NT | NT | NT | NT | NT | NT | NT | S | S | NT | S | NT | NT | S | NT | NT | NT | NT | NT | NT | NT | NT |
| **ID** | **Pathogen** | **Ceft** | **Cefu** | **Gent** | **cipro** | **Neti** | **Aug** | **cotri** | **amik** | **mero** | **eryth** | **clind** | **cefo** | **amp** | **Ceftaz** | **Cef_sul** | **pen** | **col** | **vanc** | **fusi** | **pip** | **lin** | **teico** | **fluco** | **ampho** |
| 48912 | Candida albicans | NT | NT | NT | NT | NT | NT | NT | NT | NT | NT | NT | NT | NT | NT | NT | NT | NT | NT | NT | NT | NT | NT | S | S |
| 49614 | E. Coli | R | R | S | NT | NT | R | S | S | S | NT | NT | NT | R | NT | NT | NT | NT | NT | NT | NT | NT | NT | NT | NT |
| 50541 | Enterococcus faecium | NT | NT | NT | NT | NT | NT | NT | NT | NT | NT | NT | NT | R | NT | NT | NT | NT | S | NT | NT | S | S | NT | NT |
| 50542 | Enterobacter cloacae | R | R | R | R | NT | R | R | S | R | NT | NT | NT | NT | R | R | NT | NT | NT | NT | NT | NT | NT | NT | NT |
| 50876 | GBS | NT | NT | NT | NT | NT | NT | NT | NT | NT | NT | R | NT | S | NT | NT | S | NT | NT | NT | NT | NT | NT | NT | NT |
| 51794 | E. Coli | R | R | S | NT | NT | R | S | S | S | NT | NT | NT | R | NT | NT | NT | NT | NT | NT | NT | NT | NT | NT | NT |
| 52205 | GBS | NT | NT | NT | NT | NT | NT | NT | NT | NT | NT | R | NT | S | NT | NT | S | NT | NT | NT | NT | NT | NT | NT | NT |
| 52450 | E. Coli | R | R | S | NT | NT | R | S | S | S | NT | NT | NT | R | NT | NT | NT | NT | NT | NT | NT | NT | NT | NT | NT |
| 54814 | S. aureus | NT | NT | S | S | NT | NT | S | NT | NT | NT | NT | NT | NT | NT | NT | NT | NT | NT | NT | NT | NT | NT | NT | NT |
| 55339 | NLF Coliform | R | R | R | R | R | R | R | R | R | NT | NT | NT | R | NT | NT | NT | NT | NT | NT | NT | NT | NT | NT | NT |
| 55751 | NLF Coliform | R | R | R | R | R | R | R | R | R | NT | NT | NT | R | NT | NT | NT | NT | NT | NT | NT | NT | NT | NT | NT |
| 55804 | NLF Coliform | R | R | R | R | R | R | R | R | R | NT | NT | NT | R | NT | NT | NT | NT | NT | NT | NT | NT | NT | NT | NT |
| 55809 | NLF Coliform | R | R | R | R | R | R | R | S | R | NT | NT | NT | R | NT | NT | NT | NT | NT | NT | NT | NT | NT | NT | NT |
| 56128 | Acinetobacter sp. | R | R | NT | NT | NT | R | NT | R | R | NT | NT | NT | R | NT | S | NT | NT | NT | NT | NT | NT | NT | NT | NT |
| 56463 | NLF Coliform | R | R | R | R | R | R | R | S | R | NT | NT | NT | R | NT | NT | NT | NT | NT | NT | NT | NT | NT | NT | NT |
| 57552 | NLF Coliform | R | R | R | R | R | R | R | S | R | NT | NT | NT | R | NT | NT | NT | NT | NT | NT | NT | NT | NT | NT | NT |
| 57821 | Candida parapsilosis | NT | NT | NT | NT | NT | NT | NT | NT | NT | NT | NT | NT | NT | NT | NT | NT | NT | NT | NT | NT | NT | NT | S | S |
| 57900 | Candida parapsilosis | NT | NT | NT | NT | NT | NT | NT | NT | NT | NT | NT | NT | NT | NT | NT | NT | NT | NT | NT | NT | NT | NT | S | S |
| 57900 | E. faecium | R | R | R | NT | NT | NT | NT | NT | NT | NT | NT | NT | R | NT | NT | NT | NT | R | NT | NT | S | S | NT | NT |
| 59025 | E. Coli | R | R | S | S | NT | R | R | S | S | NT | NT | NT | R | NT | NT | NT | NT | NT | NT | NT | NT | NT | NT | NT |
| 59025 | K. pneumoniae | R | R | R | R | NT | R | R | S | S | NT | NT | NT | R | NT | NT | NT | NT | NT | NT | NT | NT | NT | NT | NT |
| 60019 | Acinetobacter sp. | R | R | R | NT | R | R | R | R | R | NT | NT | NT | R | R | NT | NT | NT | NT | NT | NT | NT | NT | NT | NT |
| 60750 | LF Coliform | S | S | S | S | S | S | S | S | S | NT | NT | NT | R | NT | NT | NT | NT | NT | NT | NT | NT | NT | NT | NT |
| 63391 | Acinetobacter sp. | R | R | R | R | R | R | R | R | R | NT | NT | NT | R | R | NT | NT | NT | NT | NT | NT | NT | NT | NT | NT |
| 64466 | Acinetobacter sp. | NT | NT | R | R | R | NT | S | R | R | NT | NT | NT | NT | R | NT | NT | S | NT | NT | NT | NT | NT | NT | NT |
| 64466 | Enterobacter cloacae | R | R | R | R | NT | R | R | S | R | NT | NT | NT | R | R | NT | NT | NT | NT | NT | NT | NT | NT | NT | NT |
| 64863 | S. aureus | NT | NT | S | S | NT | NT | S | NT | NT | S | S | R | NT | NT | NT | NT | NT | NT | NT | NT | NT | NT | NT | NT |
| **ID** | **Pathogen** | **Ceft** | **Cefu** | **Gent** | **cipro** | **Neti** | **Aug** | **cotri** | **amik** | **mero** | **eryth** | **clind** | **cefo** | **amp** | **Ceftaz** | **Cef_sul** | **pen** | **col** | **vanc** | **fusi** | **pip** | **lin** | **teico** | **fluco** | **ampho** |
| 64883 | GBS | S | NT | NT | NT | NT | NT | NT | NT | NT | R | R | NT | S | NT | NT | S | NT | NT | NT | NT | NT | NT | NT | NT |
| 66045 | LF Coliform | R | R | R | R | R | R | R | R | R | NT | NT | NT | R | NT | NT | NT | NT | NT | NT | NT | NT | NT | NT | NT |
| 66380 | Acinetobacter sp. | R | R | R | R | R | R | R | R | R | NT | NT | NT | R | R | NT | NT | NT | NT | NT | NT | NT | NT | NT | NT |
| 67106 | NLF Coliform | S | S | S | S | S | S | S | S | S | NT | NT | NT | S | NT | NT | NT | NT | NT | NT | NT | NT | NT | NT | NT |
| 67107 | LF Coliform | R | R | R | R | R | R | R | R | R | NT | NT | NT | R | NT | NT | NT | NT | NT | NT | NT | NT | NT | NT | NT |
| 71156 | Pseudomonas sp. | NT | R | S | S | S | S | R | S | S | NT | NT | NT | NT | R | NT | NT | NT | NT | NT | NT | NT | NT | NT | NT |
| 71388 | Enterobacter cloacae | R | R | R | R | S | R | R | S | R | NT | NT | NT | NT | NT | NT | NT | NT | NT | NT | NT | NT | NT | NT | NT |
| 72531 | Acinetobacter sp. | R | R | R | R | R | R | R | R | R | NT | NT | NT | R | R | NT | NT | NT | NT | NT | NT | NT | NT | NT | NT |
| 72950 | NLF Coliform | R | R | R | R | R | R | R | R | R | NT | NT | NT | R | R | R | NT | NT | NT | NT | NT | NT | NT | NT | NT |
| 73280 | GBS | NT | NT | NT | NT | NT | NT | NT | NT | NT | S | S | NT | S | NT | NT | S | NT | NT | NT | NT | NT | NT | NT | NT |
| 73293 | Acinetobacter sp. | NT | NT | R | R | S | R | R | S | R | NT | NT | NT | NT | NT | NT | NT | NT | NT | NT | NT | NT | NT | NT | NT |
| 74025 | Acinetobacter sp. | R | R | R | R | R | R | R | R | R | NT | NT | NT | NT | NT | NT | NT | NT | NT | NT | NT | NT | NT | NT | NT |
| 74495 | GBS | NT | NT | NT | NT | NT | NT | NT | NT | NT | R | R | NT | S | NT | NT | S | NT | NT | NT | NT | NT | NT | NT | NT |
| 75122 | Enterobacter cloacae | R | R | R | R | R | R | R | S | R | NT | NT | NT | R | R | R | NT | NT | NT | NT | NT | NT | NT | NT | NT |
| 76214 | LF Coliform | R | R | R | R | R | R | R | R | R | NT | NT | NT | R | R | R | NT | NT | NT | NT | NT | NT | NT | NT | NT |
| 76225 | Acinetobacter sp. | R | R | R | R | R | R | R | R | R | NT | NT | NT | R | R | NT | NT | NT | NT | NT | NT | NT | NT | NT | NT |
| 77379 | LF Coliform | R | R | R | R | R | R | R | S | R | NT | NT | NT | R | R | R | NT | NT | NT | NT | NT | NT | NT | NT | NT |
| 78013 | LF Coliform | R | R | R | S | NT | R | NT | S | S | NT | NT | NT | R | NT | S | NT | NT | NT | NT | NT | NT | NT | NT | NT |
| 78760 | GBS | S | NT | NT | NT | NT | NT | NT | NT | NT | S | S | NT | S | NT | NT | S | NT | NT | NT | NT | NT | NT | NT | NT |
| 79924 | Acinetobacter sp. | R | R | R | R | R | NT | R | R | R | NT | NT | NT | R | R | NT | NT | NT | NT | NT | NT | NT | NT | NT | NT |
| 80574 | Enterococcus sp. | NT | NT | NT | NT | NT | NT | NT | NT | NT | NT | NT | NT | S | NT | NT | S | NT | NT | NT | NT | NT | NT | NT | NT |
| 80859 | NLF Coliform | S | S | R | S | S | NT | R | S | S | NT | NT | NT | S | NT | NT | NT | NT | NT | NT | NT | NT | NT | NT | NT |
| 83031 | Acinetobacter baumanii | NT | NT | R | R | R | R | R | R | R | NT | NT | NT | NT | R | R | NT | S | NT | NT | NT | NT | NT | NT | NT |
| 85726 | S. aureus | NT | NT | S | S | NT | NT | S | R | NT | R | S | R | NT | NT | NT | NT | NT | NT | NT | NT | NT | NT | NT | NT |
| 86198 | GBS | NT | NT | NT | NT | NT | NT | NT | NT | NT | S | S | NT | S | NT | NT | S | NT | NT | NT | NT | NT | NT | NT | NT |
| 89105 | NLF Coliform | R | R | R | R | R | R | R | R | S | NT | NT | NT | R | R | NT | NT | NT | NT | NT | NT | NT | NT | NT | NT |
| **ID** | **Pathogen** | **Ceft** | **Cefu** | **Gent** | **cipro** | **Neti** | **Aug** | **cotri** | **amik** | **mero** | **eryth** | **clind** | **cefo** | **amp** | **Ceftaz** | **Cef_sul** | **pen** | **col** | **vanc** | **fusi** | **pip** | **lin** | **teico** | **fluco** | **ampho** |
| 93269 | K. pneumoniae | R | R | R | R | R | R | R | S | S | NT | NT | NT | R | R | S | NT | NT | NT | NT | NT | NT | NT | NT | NT |
| 93745 | S. aureus | NT | NT | NT | S | NT | NT | S | NT | NT | R | NT | S | NT | NT | NT | NT | NT | NT | NT | NT | NT | NT | NT | NT |
| 93794 | Pseudomonas sp. | NT | NT | R | S | S | NT | R | S | S | NT | NT | NT | NT | NT | NT | NT | NT | NT | NT | NT | NT | NT | NT | NT |
| 93967 | GBS | NT | NT | NT | NT | NT | NT | NT | NT | NT | R | S | NT | S | NT | NT | S | NT | NT | NT | NT | NT | NT | NT | NT |
| 96006 | LF Coliform | R | R | R | R | R | R | R | R | R | NT | NT | NT | R | NT | NT | NT | NT | NT | NT | NT | NT | NT | NT | NT |
| 96019 | Enterobacter cloacae | R | R | R | R | R | R | R | S | R | NT | NT | NT | R | R | R | NT | NT | NT | NT | NT | NT | NT | NT | NT |
| 96771 | Acinetobacter baumanii | R | NT | NT | NT | NT | NT | R | R | R | NT | NT | NT | NT | R | NT | NT | NT | NT | NT | NT | NT | NT | NT | NT |
| 97669 | LF Coliform | R | R | R | R | R | R | R | S | R | NT | NT | NT | R | NT | NT | NT | NT | NT | NT | NT | NT | NT | NT | NT |
| 97694 | LF Coliform | R | R | S | S | NT | R | NT | R | NT | NT | NT | NT | R | R | NT | NT | NT | NT | NT | NT | NT | NT | NT | NT |
| 99142 | Enterobacter cloacae | R | R | R | R | NT | R | S | S | R | NT | NT | NT | R | NT | NT | NT | NT | NT | NT | NT | NT | NT | NT | NT |
| 101151 | LF Coliform | R | R | R | R | R | R | R | R | R | NT | NT | NT | R | NT | NT | NT | NT | NT | NT | NT | NT | NT | NT | NT |
| 101774 | Enterococcus sp. | NT | NT | NT | NT | NT | NT | NT | NT | NT | R | R | NT | S | NT | NT | NT | NT | NT | NT | NT | NT | NT | NT | NT |
| 102867 | NLF Coliform | R | R | NT | R | R | R | R | R | R | NT | NT | NT | R | R | NT | NT | NT | NT | NT | NT | NT | NT | NT | NT |
| 102868 | Acinetobacter sp. | R | R | NT | R | NT | R | R | R | R | NT | NT | NT | R | R | NT | NT | NT | NT | NT | NT | NT | NT | NT | NT |
| 103162 | Group A Strep | NT | NT | NT | NT | NT | NT | NT | NT | NT | NT | NT | NT | NT | NT | NT | S | NT | NT | NT | NT | NT | NT | NT | NT |
| 103508 | Candida krusei | NT | NT | NT | NT | NT | NT | NT | NT | NT | NT | NT | NT | NT | NT | NT | NT | NT | NT | NT | NT | NT | NT | NT | NT |
| 103508 | K. pneumoniae | R | R | S | S | NT | R | S | S | S | NT | NT | NT | R | R | NT | NT | NT | NT | NT | NT | NT | NT | NT | NT |
| 104583 | Acinetobacter sp. | R | R | R | R | R | R | R | R | R | NT | NT | NT | NT | NT | NT | NT | NT | NT | NT | NT | NT | NT | NT | NT |
| 105827 | E. Coli | R | R | S | S | NT | R | R | S | S | NT | NT | NT | R | R | NT | NT | NT | NT | NT | NT | NT | NT | NT | NT |
| 108468 | K. pneumoniae | R | NT | S | R | NT | R | S | S | R | NT | NT | NT | R | R | S | NT | NT | NT | NT | NT | NT | NT | NT | NT |
| 110692 | E. Coli | S | S | S | S | S | S | NT | S | S | NT | NT | NT | S | NT | NT | NT | NT | NT | NT | NT | NT | NT | NT | NT |
| 111105 | S. aureus | NT | NT | R | R | NT | NT | R | NT | NT | NT | NT | R | NT | NT | NT | NT | NT | NT | NT | NT | NT | NT | NT | NT |
| 111648 | Enterobacter aerogenes | S | NT | S | S | S | R | S | S | S | NT | NT | NT | R | S | NT | NT | NT | NT | NT | NT | NT | NT | NT | NT |
| 112258 | LF Coliform | R | R | R | R | R | R | R | R | R | NT | NT | NT | R | NT | S | NT | NT | NT | NT | NT | NT | NT | NT | NT |
| 114966 | GBS | NT | NT | NT | NT | NT | NT | NT | NT | NT | NT | NT | NT | S | NT | NT | S | NT | NT | NT | NT | NT | NT | NT | NT |
| 115422 | Candida sp. | NT | NT | NT | NT | NT | NT | NT | NT | NT | NT | NT | NT | NT | NT | NT | NT | NT | NT | NT | NT | NT | NT | NT | NT |
| **ID** | **Pathogen** | **Ceft** | **Cefu** | **Gent** | **cipro** | **Neti** | **Aug** | **cotri** | **amik** | **mero** | **eryth** | **clind** | **cefo** | **amp** | **Ceftaz** | **Cef_sul** | **pen** | **col** | **vanc** | **fusi** | **pip** | **lin** | **teico** | **fluco** | **ampho** |
| 115422 | Enterobacter cloacae | R | R | R | R | NT | R | R | S | R | NT | NT | NT | R | R | S | NT | NT | NT | NT | NT | NT | NT | NT | NT |
| 116742 | GBS | NT | NT | NT | NT | NT | NT | NT | NT | NT | S | S | NT | S | NT | NT | S | NT | NT | NT | NT | NT | NT | NT | NT |
| 117506 | Serratia marcescens | R | R | R | R | R | R | R | S | R | NT | NT | NT | R | R | NT | NT | NT | NT | NT | NT | NT | NT | NT | NT |
| 117664 | NLF Coliform | R | R | R | R | S | R | NT | S | S | NT | NT | NT | R | NT | NT | NT | NT | NT | NT | NT | NT | NT | NT | NT |
| 118084 | Enterobacter cloacae | R | R | R | R | NT | NT | R | S | S | NT | NT | NT | R | R | NT | NT | S | NT | NT | NT | NT | NT | NT | NT |
| 120349 | LF Coliform | R | R | R | R | R | R | S | R | R | NT | NT | NT | R | R | NT | NT | NT | NT | NT | NT | NT | NT | NT | NT |
| 120349 | Acinetobacter baumanii | R | R | S | S | S | S | S | S | S | NT | NT | NT | R | S | NT | NT | NT | NT | NT | NT | NT | NT | NT | NT |
| 122566 | Enterobacter cloacae | NT | NT | R | R | NT | NT | S | R | R | NT | NT | NT | NT | R | S | NT | NT | NT | NT | NT | NT | NT | NT | NT |
| 123232 | NLF Coliform | S | NT | S | S | NT | NT | R | S | S | NT | NT | NT | NT | NT | S | NT | NT | NT | NT | NT | NT | NT | NT | NT |
| 125145 | Acinetobacter sp. | R | NT | R | R | NT | R | R | R | R | NT | NT | NT | R | R | S | NT | NT | NT | NT | NT | NT | NT | NT | NT |
| 125218 | GBS | S | NT | NT | NT | NT | NT | NT | NT | NT | S | S | NT | S | NT | NT | S | NT | NT | NT | NT | NT | NT | NT | NT |
| 125397 | Acinetobacter sp. | R | R | R | R | R | R | S | R | R | NT | NT | NT | NT | R | NT | NT | NT | NT | NT | NT | NT | NT | NT | NT |
| 125397 | LF Coliform | R | R | R | R | S | R | R | R | R | NT | NT | NT | R | R | NT | NT | NT | NT | NT | NT | NT | NT | NT | NT |
| 125398 | Acinetobacter sp. | R | R | R | R | R | R | S | R | R | NT | NT | NT | NT | R | NT | NT | NT | NT | NT | NT | NT | NT | NT | NT |
| 125398 | LF Coliform | R | R | R | R | S | R | R | R | R | NT | NT | NT | R | R | NT | NT | NT | NT | NT | NT | NT | NT | NT | NT |
| 125543 | Enterobacter cloacae | R | R | R | R | NT | R | R | S | R | NT | NT | NT | R | R | S | NT | NT | NT | NT | NT | NT | NT | NT | NT |
| 125543 | NLF Coliform | R | R | R | R | NT | R | R | S | R | NT | NT | NT | R | NT | NT | NT | NT | NT | NT | NT | NT | NT | NT | NT |
| 127367 | Serratia marcescens | R | R | R | R | NT | R | S | S | S | NT | NT | NT | R | R | NT | NT | NT | NT | NT | S | NT | NT | NT | NT |
| 130146 | LF Coliform | R | R | S | S | R | R | S | R | R | NT | NT | NT | R | R | NT | NT | NT | NT | NT | NT | NT | NT | NT | NT |
| 133855 | Candida guilliermondii | NT | NT | NT | NT | NT | NT | NT | NT | NT | NT | NT | NT | NT | NT | NT | NT | NT | NT | NT | NT | NT | NT | S | S |
| 134071 | K. pneumoniae | R | R | S | NT | NT | R | S | S | S | NT | NT | NT | R | R | NT | NT | NT | NT | NT | NT | NT | NT | NT | NT |
| 135855 | Pseudomonas sp. | NT | NT | S | S | R | R | S | S | S | NT | NT | NT | NT | R | NT | NT | NT | NT | NT | NT | NT | NT | NT | NT |
| 137284 | Enterobacter cloacae | R | R | R | R | NT | R | R | S | R | NT | NT | NT | NT | R | NT | NT | NT | NT | NT | NT | NT | NT | NT | NT |
| 138659 | NLF Coliform | R | R | R | R | R | R | R | NT | R | NT | NT | NT | R | R | S | NT | NT | NT | NT | NT | NT | NT | NT | NT |
| 147386 | Acinetobacter sp. | R | R | R | R | NT | R | NT | R | R | NT | NT | NT | R | R | S | NT | NT | NT | NT | NT | NT | NT | NT | NT |
|  |  |  |  |  |  |  |  |  |  |  |  |  |  |  |  |  |  |  |  |  |  |  |  |  |  |
| **ID** | **Pathogen** | **Ceft** | **Cefu** | **Gent** | **cipro** | **Neti** | **Aug** | **cotri** | **amik** | **mero** | **eryth** | **clind** | **cefo** | **amp** | **Ceftaz** | **Cef_sul** | **pen** | **col** | **vanc** | **fusi** | **pip** | **lin** | **teico** | **fluco** | **ampho** |
| 149803 | LF Coliform | R | R | R | R | R | R | R | S | S | NT | NT | NT | NT | NT | NT | NT | NT | NT | NT | NT | NT | NT | NT | NT |
| 155307 | LF Coliform | R | R | R | S | NT | R | NT | S | S | NT | NT | NT | R | NT | NT | NT | NT | NT | NT | NT | NT | NT | NT | NT |
| 155307 | NLF Coliform | R | R | NT | S | NT | R | NT | S | S | NT | NT | NT | R | NT | NT | NT | NT | NT | NT | NT | NT | NT | NT | NT |
|  | GBS | NT | NT | NT | NT | NT | NT | NT | NT | NT | S | S | NT | S | NT | NT | S | NT | NT | NT | NT | NT | NT | NT | NT |
| 15697 | GBS | S | NT | NT | NT | NT | NT | NT | NT | NT | NT | NT | NT | NT | NT | NT | S | NT | NT | NT | NT | NT | NT | NT | NT |
| 22109 | NLF Coliform | R | R | R | NT | R | NT | NT | R | NT | NT | NT | NT | NT | NT | NT | NT | NT | NT | NT | NT | NT | NT | NT | NT |
| 27543 | GBS | S | NT | NT | NT | NT | NT | NT | NT | NT | NT | NT | NT | NT | NT | NT | S | NT | NT | NT | NT | NT | NT | NT | NT |
| 39922 | GBS | S | NT | NT | NT | NT | NT | NT | NT | NT | NT | NT | NT | NT | NT | NT | S | NT | NT | NT | NT | NT | NT | NT | NT |
| 34600 | LF Coliform | R | R | R | R | R | NT | NT | R | NT | NT | NT | NT | NT | NT | NT | NT | NT | NT | NT | NT | NT | NT | NT | NT |
| 33633 | Acinetobacter sp. | NT | NT | NT | R | R | NT | S | R | NT | NT | NT | NT | NT | NT | NT | NT | NT | NT | NT | NT | NT | NT | NT | NT |
| 38173 | NLF Coliform | R | R | NT | R | NT | NT | NT | R | NT | NT | NT | NT | NT | NT | NT | NT | NT | NT | NT | NT | NT | NT | NT | NT |
| 38173 | Acinetobacter sp. | NT | NT | S | S | NT | NT | NT | S | NT | NT | NT | NT | NT | NT | NT | NT | NT | NT | NT | NT | NT | NT | NT | NT |
| 47370 | NLF Coliform | R | R | S | S | S | NT | NT | S | NT | NT | NT | NT | NT | NT | NT | NT | NT | NT | NT | NT | NT | NT | NT | NT |
| 48300 | NLF Coliform | S | S | NT | S | S | NT | NT | S | NT | NT | NT | NT | NT | NT | NT | NT | NT | NT | NT | NT | NT | NT | NT | NT |
| 95046 | GBS | S | NT | NT | NT | NT | NT | NT | NT | NT | NT | NT | NT | NT | NT | NT | S | NT | NT | NT | NT | NT | NT | NT | NT |
| 103501 | GBS | S | NT | NT | NT | NT | NT | NT | NT | NT | NT | NT | NT | S | NT | NT | S | NT | NT | NT | NT | NT | NT | NT | NT |
| 110277 | GBS | S | NT | NT | NT | NT | NT | NT | NT | NT | NT | NT | NT | S | NT | NT | S | NT | NT | NT | NT | NT | NT | NT | NT |
| 110145 | S. pneumoniae | S | NT | NT | NT | NT | NT | NT | NT | NT | NT | NT | NT | NT | NT | NT | S | NT | S | NT | NT | NT | NT | NT | NT |
| 112095 | Group A Strep | NT | NT | NT | NT | NT | NT | NT | NT | NT | NT | NT | NT | NT | NT | NT | S | NT | NT | NT | NT | NT | NT | NT | NT |
| 112706 | Acinetobacter sp. | NT | NT | R | NT | R | NT | NT | R | NT | NT | NT | NT | NT | NT | NT | NT | NT | NT | NT | NT | NT | NT | NT | NT |
| 115374 | Acinetobacter sp. | NT | NT | R | NT | R | NT | NT | R | NT | NT | NT | NT | NT | NT | S | NT | NT | NT | NT | NT | NT | NT | NT | NT |
| 122224 | Acinetobacter sp. | NT | NT | S | NT | S | NT | NT | S | NT | NT | NT | NT | NT | NT | NT | NT | NT | NT | NT | NT | NT | NT | NT | NT |
| 123732 | Acinetobacter sp. | NT | NT | R | NT | R | NT | NT | R | NT | NT | NT | NT | NT | NT | R | NT | NT | NT | NT | NT | NT | NT | NT | NT |
| 124103 | Acinetobacter sp. | NT | NT | S | NT | S | NT | NT | S | NT | NT | NT | NT | NT | NT | S | NT | NT | NT | NT | NT | NT | NT | NT | NT |
| 122274 | Acinetobacter sp. | NT | NT | S | NT | NT | NT | NT | S | NT | NT | NT | NT | NT | NT | NT | NT | NT | NT | NT | NT | NT | NT | NT | NT |
|  |  |  |  |  |  |  |  |  |  |  |  |  |  |  |  |  |  |  |  |  |  |  |  |  |  |
| **ID** | **Pathogen** | **Ceft** | **Cefu** | **Gent** | **cipro** | **Neti** | **Aug** | **cotri** | **amik** | **mero** | **eryth** | **clind** | **cefo** | **amp** | **Ceftaz** | **Cef_sul** | **pen** | **col** | **vanc** | **fusi** | **pip** | **lin** | **teico** | **fluco** | **ampho** |
| 127552 | Group A Strep | NT | NT | NT | NT | NT | NT | NT | NT | NT | NT | NT | NT | NT | NT | NT | S | NT | NT | NT | NT | NT | NT | NT | NT |
| 16946 | GBS | S | NT | NT | NT | NT | NT | NT | NT | NT | NT | NT | NT | S | NT | NT | S | NT | NT | NT | NT | NT | NT | NT | NT |
| 31367 | NLF Coliform | R | R | R | NT | R | NT | NT | R | NT | NT | NT | NT | NT | NT | NT | NT | NT | NT | NT | NT | NT | NT | NT | NT |
| 34212 | Acinetobacter sp. | NT | NT | R | NT | R | NT | NT | R | NT | NT | NT | NT | NT | NT | R | NT | NT | NT | NT | NT | NT | NT | NT | NT |
| 38191 | GBS | S | NT | NT | NT | NT | NT | NT | NT | NT | NT | NT | NT | S | NT | NT | S | NT | NT | NT | NT | NT | NT | NT | NT |
| 48271 | GBS | S | NT | NT | NT | NT | NT | NT | NT | NT | NT | NT | NT | S | NT | NT | S | NT | NT | NT | NT | NT | NT | NT | NT |
| 49020 | Pseudomonas sp. | NT | NT | R | NT | NT | NT | NT | NT | NT | NT | NT | NT | NT | NT | NT | NT | NT | NT | NT | NT | NT | NT | NT | NT |
| 62312 | Pseudomonas sp. | NT | NT | S | NT | NT | NT | NT | NT | NT | NT | NT | NT | NT | NT | NT | NT | NT | NT | NT | NT | NT | NT | NT | NT |
| 63473 | GBS | S | NT | NT | NT | NT | NT | NT | NT | NT | NT | NT | NT | S | NT | NT | S | NT | NT | NT | NT | NT | NT | NT | NT |
| 64650 | LF Coliform | R | R | NT | S | S | NT | NT | S | NT | NT | NT | NT | NT | NT | NT | NT | NT | NT | NT | NT | NT | NT | NT | NT |
| 67957 | K. pneumoniae | R | R | S | S | S | NT | NT | NT | NT | NT | NT | NT | NT | NT | NT | NT | NT | NT | NT | NT | NT | NT | NT | NT |
| 69167 | Enterobacter cloacae | R | R | R | S | S | NT | NT | NT | NT | NT | NT | NT | NT | NT | NT | NT | NT | NT | NT | NT | NT | NT | NT | NT |
| 76248 | GBS | S | NT | NT | NT | NT | NT | NT | NT | NT | NT | NT | NT | S | NT | NT | S | NT | NT | NT | NT | NT | NT | NT | NT |
| 82817 | GBS | S | NT | NT | NT | NT | NT | NT | NT | NT | NT | NT | NT | S | NT | NT | S | NT | NT | NT | NT | NT | NT | NT | NT |
| 89316 | Candida sp. | NT | NT | NT | NT | NT | NT | NT | NT | NT | NT | NT | NT | NT | NT | NT | NT | NT | NT | NT | NT | NT | NT | NT | NT |
| 95675 | LF Coliform | S | S | R | R | S | NT | NT | S | NT | NT | NT | NT | NT | NT | NT | NT | NT | NT | NT | NT | NT | NT | NT | NT |
| 95675 | Candida sp. | NT | NT | NT | NT | NT | NT | NT | NT | NT | NT | NT | NT | NT | NT | NT | NT | NT | NT | NT | NT | NT | NT | NT | NT |
| 104897 | GBS | S | NT | NT | NT | NT | NT | NT | NT | NT | NT | NT | NT | S | NT | NT | S | NT | NT | NT | NT | NT | NT | NT | NT |
| 110668 | Enterobacter cloacae | R | NT | NT | S | NT | NT | NT | S | NT | NT | NT | NT | NT | NT | NT | NT | NT | NT | NT | NT | NT | NT | NT | NT |
| 112563 | S. pneumoniae | S | NT | NT | NT | NT | NT | NT | NT | NT | NT | NT | NT | NT | NT | NT | S | NT | S | NT | NT | NT | NT | NT | NT |
| 108153 | LF Coliform | R | R | S | NT | S | NT | NT | S | NT | NT | NT | NT | NT | NT | NT | NT | NT | NT | NT | NT | NT | NT | NT | NT |
| 118108 | GBS | S | NT | NT | NT | NT | NT | NT | NT | NT | NT | NT | NT | S | NT | NT | S | NT | NT | NT | NT | NT | NT | NT | NT |
| 117652 | Acinetobacter sp. | NT | NT | R | R | R | NT | NT | R | NT | NT | NT | NT | NT | NT | NT | NT | NT | NT | NT | NT | NT | NT | NT | NT |
| 117652 | Candida sp. | NT | NT | NT | NT | NT | NT | NT | NT | NT | NT | NT | NT | NT | NT | NT | NT | NT | NT | NT | NT | NT | NT | NT | NT |
| 117757 | Klebsiella sp. | NT | NT | S | S | S | NT | NT | S | NT | NT | NT | NT | NT | NT | NT | NT | NT | NT | NT | NT | NT | NT | NT | NT |
| 119340 | Candida sp. | NT | NT | NT | NT | NT | NT | NT | NT | NT | NT | NT | NT | NT | NT | NT | NT | NT | NT | NT | NT | NT | NT | NT | NT |
| 131434 | LF Coliform | NT | NT | R | S | S | NT | NT | NT | NT | NT | NT | NT | NT | NT | NT | NT | NT | NT | NT | NT | NT | NT | NT | NT |
| **ID** | **Pathogen** | **Ceft** | **Cefu** | **Gent** | **cipro** | **Neti** | **Aug** | **cotri** | **amik** | **mero** | **eryth** | **clind** | **cefo** | **amp** | **Ceftaz** | **Cef_sul** | **pen** | **col** | **vanc** | **fusi** | **pip** | **lin** | **teico** | **fluco** | **ampho** |
| 119254 | Candida parapsilosis | NT | NT | NT | NT | NT | NT | NT | NT | NT | NT | NT | NT | NT | NT | NT | NT | NT | NT | NT | NT | NT | NT | S | S |
| 134205 | Candida parapsilosis | NT | NT | NT | NT | NT | NT | NT | NT | NT | NT | NT | NT | NT | NT | NT | NT | NT | NT | NT | NT | NT | NT | S | S |
| 24658 | GBS | S | NT | NT | NT | NT | NT | NT | NT | NT | NT | NT | NT | S | NT | NT | S | NT | NT | NT | NT | NT | NT | NT | NT |
| 31299 | Enterococcus sp. | NT | NT | NT | NT | NT | NT | NT | NT | NT | NT | NT | NT | S | NT | NT | NT | NT | NT | NT | NT | NT | NT | NT | NT |
| 33750 | Pseudomonas sp. | NT | NT | NT | R | R | NT | NT | NT | NT | NT | NT | NT | NT | NT | NT | NT | NT | NT | NT | NT | NT | NT | NT | NT |
| 42527 | NLF Coliform | S | S | S | S | S | S | NT | NT | NT | NT | NT | NT | NT | NT | NT | NT | NT | NT | NT | NT | NT | NT | NT | NT |
| 42918 | Acinetobacter sp. | NT | NT | R | R | R | R | NT | NT | NT | NT | NT | NT | NT | NT | NT | NT | NT | NT | NT | NT | NT | NT | NT | NT |
| 63264 | Acinetobacter sp. | NT | NT | R | R | R | R | NT | NT | NT | NT | NT | NT | NT | NT | S | NT | NT | NT | NT | NT | NT | NT | NT | NT |
| 63244 | Acinetobacter sp. | NT | NT | S | S | S | R | NT | NT | NT | NT | NT | NT | NT | NT | S | NT | NT | NT | NT | NT | NT | NT | NT | NT |
| 62036 | LF Coliform | R | R | R | R | R | R | NT | NT | NT | NT | NT | NT | NT | NT | NT | NT | NT | NT | NT | NT | NT | NT | NT | NT |
| 62685 | S. aureus | S | NT | S | S | S | NT | NT | NT | NT | NT | S | S | S | NT | NT | NT | NT | NT | NT | NT | NT | NT | NT | NT |
| 65660 | LF Coliform | R | R | R | S | S | R | NT | NT | NT | NT | NT | NT | NT | NT | NT | NT | NT | NT | NT | NT | NT | NT | NT | NT |
| 63244 | Pseudomonas sp. | NT | NT | R | R | R | NT | NT | NT | NT | NT | NT | NT | NT | NT | NT | NT | NT | NT | NT | NT | NT | NT | NT | NT |
| 73732 | NLF Coliform | R | R | R | S | S | R | NT | S | NT | NT | NT | NT | NT | NT | NT | NT | NT | NT | NT | NT | NT | NT | NT | NT |
| 84788 | NLF Coliform | S | S | S | S | S | S | NT | S | NT | NT | NT | NT | R | NT | NT | NT | NT | NT | NT | NT | NT | NT | NT | NT |
| 90628 | NLF Coliform | R | R | R | S | S | S | NT | S | NT | NT | NT | NT | NT | NT | NT | NT | NT | NT | NT | NT | NT | NT | NT | NT |
| 90628 | LF Coliform | R | R | NT | S | S | S | NT | S | NT | NT | NT | NT | NT | NT | NT | NT | NT | NT | NT | NT | NT | NT | NT | NT |
| 102757 | Haemophilus aphrophilus | S | NT | S | R | S | S | NT | S | NT | NT | NT | NT | NT | NT | NT | NT | NT | NT | NT | NT | NT | NT | NT | NT |
| 103449 | GBS | S | NT | NT | NT | NT | NT | NT | NT | NT | NT | NT | NT | S | NT | NT | S | NT | NT | NT | NT | NT | NT | NT | NT |
| 102942 | LF Coliform | R | R | R | R | R | R | NT | R | NT | NT | NT | NT | NT | NT | NT | NT | NT | NT | NT | NT | NT | NT | NT | NT |
| 104228 | Candida parapsilosis | NT | NT | NT | NT | NT | NT | NT | NT | NT | NT | NT | NT | NT | NT | NT | NT | NT | NT | NT | NT | NT | NT | S | S |
| 107212 | Pseudomonas sp. | NT | NT | R | R | S | NT | NT | R | NT | NT | NT | NT | NT | NT | R | NT | NT | NT | NT | NT | NT | NT | NT | NT |
| 104228 | LF Coliform | R | R | R | S | R | R | NT | S | NT | NT | NT | NT | NT | NT | NT | NT | NT | NT | NT | NT | NT | NT | NT | NT |
| 111391 | GBS | S | NT | NT | NT | NT | NT | NT | NT | NT | NT | NT | NT | S | NT | NT | S | NT | NT | NT | NT | NT | NT | NT | NT |
| 111356 | S. aureus | NT | NT | S | S | NT | NT | NT | NT | NT | S | S | NT | NT | NT | NT | R | NT | NT | NT | NT | NT | NT | NT | NT |
| 116055 | Rhizobium radiobacter | NT | NT | R | S | S | S | NT | S | NT | NT | NT | NT | NT | NT | S | NT | NT | NT | NT | NT | NT | NT | NT | NT |
| **ID** | **Pathogen** | **Ceft** | **Cefu** | **Gent** | **cipro** | **Neti** | **Aug** | **cotri** | **amik** | **mero** | **eryth** | **clind** | **cefo** | **amp** | **Ceftaz** | **Cef_sul** | **pen** | **col** | **vanc** | **fusi** | **pip** | **lin** | **teico** | **fluco** | **ampho** |
| 115038 | Rhizobium radiobacter | NT | NT | R | S | S | S | NT | S | NT | NT | NT | NT | NT | NT | S | NT | NT | NT | NT | NT | NT | NT | NT | NT |
| 115878 | Rhizobium radiobacter | NT | NT | R | S | S | NT | NT | S | NT | NT | NT | NT | NT | NT | S | NT | NT | NT | NT | NT | NT | NT | NT | NT |
| 119384 | Rhizobium radiobacter | NT | NT | NT | NT | NT | NT | NT | NT | NT | NT | NT | NT | NT | NT | NT | NT | NT | NT | NT | NT | NT | NT | NT | NT |
| 119406 | Rhizobium radiobacter |  | NT | NT | NT | NT | NT | NT | NT | NT | NT | NT | NT | NT | NT | NT | NT | NT | NT | NT | NT | NT | NT | NT | NT |
| 122101 | GBS | S | NT | NT | NT | NT | NT | NT | NT | NT | NT | NT | NT | S | NT | NT | S | NT | NT | NT | NT | NT | NT | NT | NT |
| 121327 | Rhizobium radiobacter | NT | NT | NT | NT | NT | NT | NT | NT | NT | NT | NT | NT | NT | NT | NT | NT | NT | NT | NT | NT | NT | NT | NT | NT |
| 122523 | S. pneumoniae | S | NT | NT | NT | NT | NT | NT | NT | NT | S | S | NT | S | NT | NT | NT | NT | NT | NT | NT | NT | NT | NT | NT |
| 116055 | Candida guilliermondii | NT | NT | NT | NT | NT | NT | NT | NT | NT | NT | NT | NT | NT | NT | NT | NT | NT | NT | NT | NT | NT | NT | NT | NT |
| 123850 | S. aureus | NT | NT | S | S | NT | NT | NT | NT | NT | S | S | S | S | NT | NT | R | NT | NT | NT | NT | NT | NT | NT | NT |
| 114384 | Acinetobacter sp. | NT | NT | R | R | R | R | NT | R | NT | NT | NT | NT | NT | NT | R | NT | NT | NT | NT | NT | NT | NT | NT | NT |
| 120513 | Rhizobium radiobacter | NT | NT | NT | NT | NT | NT | NT | NT | NT | NT | NT | NT | NT | NT | NT | NT | NT | NT | NT | NT | NT | NT | NT | NT |
| 122523 | Acinetobacter sp. | NT | NT | S | S | S | S | NT | S | NT | NT | NT | NT | NT | NT | S | NT | NT | NT | NT | NT | NT | NT | NT | NT |
| 118725 | Candida parapsilosis | NT | NT | NT | NT | NT | NT | NT | NT | NT | NT | NT | NT | NT | NT | NT | NT | NT | NT | NT | NT | NT | NT | S | S |
| 135311 | GBS | S | NT | NT | NT | NT | NT | NT | NT | NT | NT | NT | NT | S | NT | NT | S | NT | NT | NT | NT | NT | NT | NT | NT |
|  | Candida parapsilosis | NT | NT | NT | NT | NT | NT | NT | NT | NT | NT | NT | NT | NT | NT | NT | NT | NT | NT | NT | NT | NT | NT | S | S |
| 131972 | Candida parapsilosis | NT | NT | NT | NT | NT | NT | NT | NT | NT | NT | NT | NT | NT | NT | NT | NT | NT | NT | NT | NT | NT | NT | S | S |

*Ceft = cefotaxime, cefu = cefuroxime, gent = gentamicin, cipro = ciprofloxacin, neti = netilmicin, aug = augmentin, cotri = cotrimoxazole, amik = amikacin, mero = meropenem, eryth = erythromycin, clind = clindamycin, cefo = cefoxitin, amp = ampicillin, ceftaz = ceftaz avibactam, cef_sul = cefoper sulbac, pen = penicillin, col = colostin, vanc = vancomycin, fusi = fusidic acid, pip = piptaz, lin = linezolid, teico = teicoplanin, fluco = fluconazole, ampho = amphotericin
