# Supplementary material for: Antimicrobial Resistance and Infant Mortality in Sri Lanka: A Retrospective Cohort Study
Source: J Paediatr Child Health. 2026 Jan 22;62(3):446–55. doi: 10.1111/jpc.70269 (PMC12976201; doi:10.1111/jpc.70269)
Supplement: Supplementary file 4 — Table S4: Antimicrobial susceptibility data, aggregated by year. [file JPC-62-446-s003.docx]

| **Supplementary Table 4: Antimicrobial susceptibility data, aggregated by Year** | | | | | | | |  |
| --- | --- | --- | --- | --- | --- | --- | --- | --- |
|  | **Year** | | | | | | |  |
|  | **2015** | **2016** | **2017** | **2018** | **2019** | **2020** | **2021** | **Total** |
| Total (N) | **22** | **26** | **42** | **44** | **62** | **22** | **33** | **251** |
| **Antibiotic** |  |  |  |  |  |  |  | |
| **Ampicillin** |  |  |  |  |  |  |  |  |
| Tested (n) | 3 | 7 | 10 | 30 | 45 | 14 | 25 | 134 |
| Sensitive (n) | 3 | 7 | 9 | 4 | 14 | 5 | 4 | 46 |
| Proportion resistant (95% CI) | 0 (0.0-0.7) | 0(0.0-0.4) | 0.1(0.003-0.4) | 0.87(0.69-0.96) | 0.69(0.53-0.82) | 0.64 (0.35-0.87) | 0.84 (0.64-0.95) | 0.66 (0.59-0.74) |
| **Gentamicin** |  |  |  |  |  |  |  |  |
| Tested (n) | 11 | 11 | 22 | 33 | 44 | 14 | 30 | 165 |
| Sensitive (n) | 6 | 4 | 8 | 6 | 13 | 6 | 9 | 52 |
| Proportion resistant (95% CI) |  | 0.64 (0.31-0.89) | 0.64(0.4-0.83) | 0.82(0.65-0.93) | 0.7(0.55-0.83) | 0.57(0.29-0.82) | 0.7(0.51-0.85) | 0.68(0.61-0.75) |
| **Cefotaxime** |  |  |  |  |  |  |  |  |
| Tested (n) | 12 | 15 | 17 | 30 | 42 | 12 | 25 | 153 |
| Sensitive (n) | 8 | 9 | 10 | 3 | 8 | 1 | 5 | 44 |
| Proportion resistant (95% CI) | 0.33 (0.09-0.65) | 0.4(0.16-0.68) | 0.4(0.18-0.67) | 0.9 (0.73-0.98) | 0.81(0.66-0.91) | 0.92(0.62-0.99) | 0.8 (0.59-0.93) | 0.71(0.63-0.78) |
| **Meropenem** |  |  |  |  |  |  |  |  |
| Tested (n) | 0 | 0 | 0 | 30 | 45 | 12 | 27 | 114 |
| Sensitive (n) | 0 | 0 | 0 | 10 | 15 | 3 | 12 | 40 |
| Proportion resistant (95% CI) | - | - | - | 0.67 (0.47-0.83) | 0.67(0.51-0.8) | 0.75(0.43-0.95) | 0.56 (0.25-0.75) | 0.65 (0.55-0.74) |

*Note: proportion resistant and confidence intervals calculated under the assumption of independence and may not account for temporal correlation within the data

Supplementary Figure 2 Gentamicin non-susceptibility by age in days as proportion with 95% CI

Supplementary Figure 3: Cefotaxime non-susceptibility by age in days as proportion with 95% CI

Supplementary Figure 4:. Meropenem non-susceptibility by age in days as proportion with 95% CI

Supplementary Figure 6:. Antimicrobial consumption and non-susceptibility by year


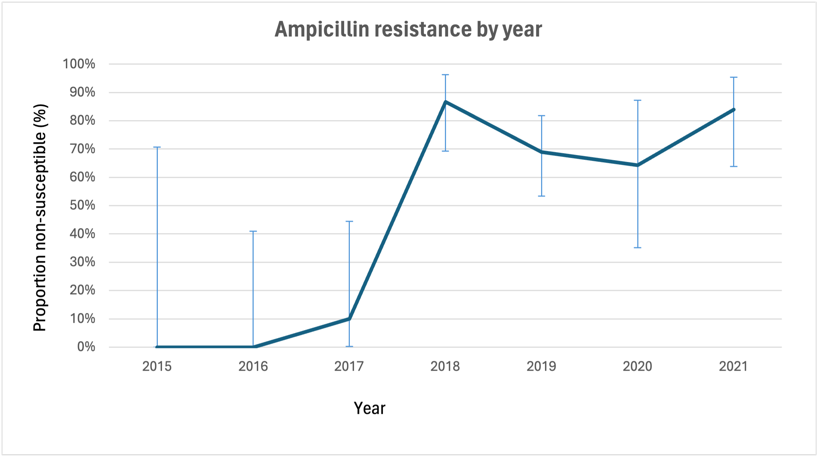

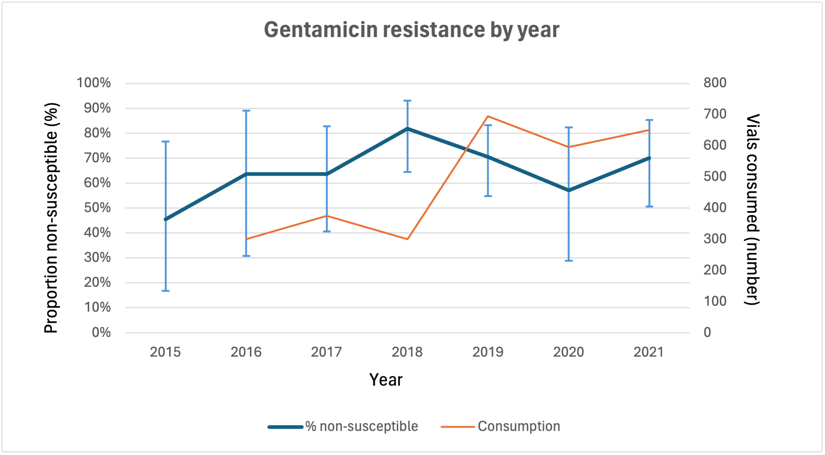

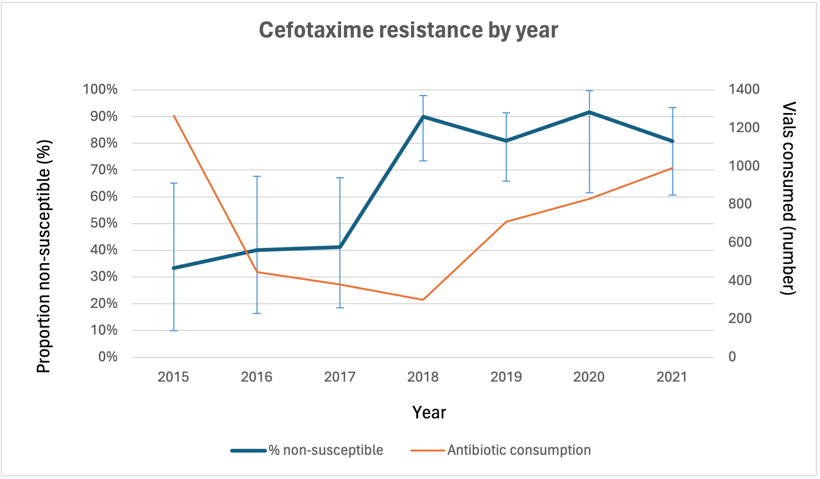

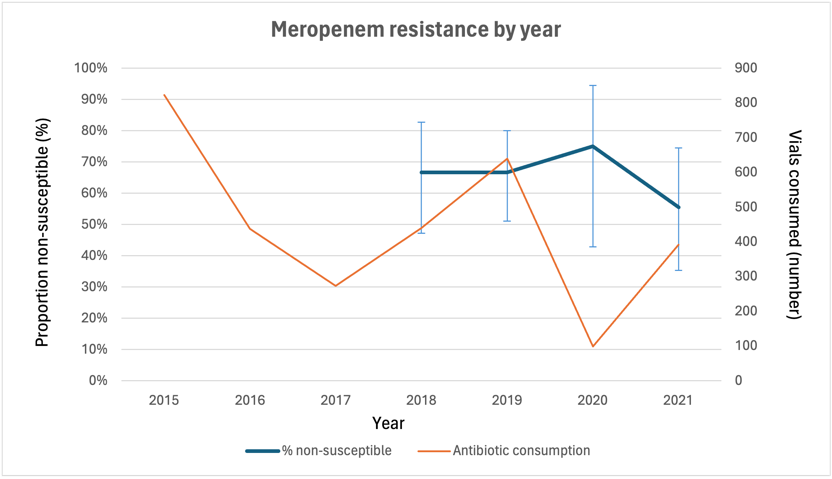


Supplementary Figure 5: Antimicrobial non-susceptibilty in Early- and late-onset sepsis
